# Maternal Antioxidant Levels in Pregnancy and Risk of Preeclampsia and Small for Gestational Age Birth: A Systematic Review and Meta-Analysis

**DOI:** 10.1371/journal.pone.0135192

**Published:** 2015-08-06

**Authors:** Jacqueline M. Cohen, Margaret Beddaoui, Michael S. Kramer, Robert W. Platt, Olga Basso, Susan R. Kahn

**Affiliations:** 1 Department of Epidemiology, Biostatistics and Occupational Health, McGill University, Montreal, Quebec, Canada; 2 Centre for Clinical Epidemiology, Lady Davis Institute for Medical Research, Jewish General Hospital, Montreal, Quebec, Canada; 3 Department of Pediatrics, McGill University, Montreal, Quebec, Canada; 4 Department of Obstetrics and Gynecology, McGill University, Montreal, Quebec, Canada; INRA, FRANCE

## Abstract

**Background:**

Oxidative stress in preeclampsia and small for gestational age (SGA) birth suggests antioxidant supplementation could prevent these conditions. However, it remains unclear whether maternal antioxidant levels are systematically lower in these pregnancies.

**Objective:**

To conduct a systematic review of the association between maternal antioxidant levels during pregnancy and preeclampsia or SGA.

**Methods:**

We searched PubMed, Embase, and several other databases from 1970–2013 for observational studies that measured maternal blood levels of non-enzymatic antioxidants (vitamins A, C, E, and carotenoids) during pregnancy or within 72 hours of delivery. The entire review process was done in duplicate. Study quality was assessed using the Newcastle-Ottawa Scale and additional questions. We pooled the standardized mean difference (SMD) across studies, stratified by outcome and pregnancy trimester, and investigated heterogeneity using meta-regression.

**Results:**

We reviewed 1,882 unique citations and 64 studies were included. Most studies were small with important risk of bias. Among studies that addressed preeclampsia (n = 58) and SGA (n = 9), 16% and 66%, respectively, measured levels prior to diagnosis. The SMDs for vitamins A, C, and E were significantly negative for overall preeclampsia, but not for mild or severe preeclampsia subtypes. Significant heterogeneity was observed in all meta-analyses and most could not be explained. Evidence for lower carotenoid antioxidants in preeclampsia and SGA was limited and inconclusive. Publication bias appears likely.

**Conclusions:**

Small, low-quality studies limit conclusions that can be drawn from the available literature. Observational studies inconsistently show that vitamins C and E or other antioxidants are lower in women who develop preeclampsia or SGA. Reverse causality remains a possible explanation for associations observed. New clinical trials are not warranted in light of this evidence; however, additional rigorous observational studies measuring antioxidant levels before clinical detection of preeclampsia and SGA may clarify whether levels are altered at a causally-relevant time of pregnancy.

## Introduction

Preeclampsia is a hypertensive disorder that affects 2–8% of pregnancies worldwide, and is most common in first pregnancies [1]. Despite remarkable progress in the understanding of the pathophysiology of preeclampsia in the last few decades, the etiology of this disorder remains unclear; a problem compounded by its heterogeneity [2]. Preeclampsia is typically diagnosed by the presence of high blood pressure and protein in the urine or adverse conditions [3–5]. It is a multisystem disorder, which can result in severe complications including seizures (eclampsia), multi-organ failure, stroke, and death [6]. Fetuses are at increased risk of preterm birth, growth restriction, and stillbirth [7]. Effective strategies for preventing preeclampsia are lacking; delivery is the only definitive cure.

Small for gestational age (SGA) is often used as a proxy for fetal growth restriction. It is defined as the birth of an infant below the 10^th^ percentile for birth weight according to a population reference, and is more common in preterm pregnancies complicated by preeclampsia [8, 9]. Infants born SGA are at increased risk of neonatal morbidity and mortality [10, 11]. SGA is associated with future growth [12], neurodevelopment, including intelligence and risk of cerebral palsy [13–15], and adult chronic disease [16].

The pathophysiology of preeclampsia and SGA are incompletely understood, but endothelial dysfunction may play a role in both conditions. Oxidative stress, caused by increased production of free radicals and insufficient antioxidant defenses, is a known cause of endothelial dysfunction, and may thus be causally related to preeclampsia and SGA [17]. Hypertension, renal disease, lupus, and older age are associated with endothelial dysfunction and are risk factors for both conditions [18, 19]. Indeed, several studies to date have shown that markers of oxidative damage are elevated and antioxidant vitamin levels are lower, in women with preeclampsia [20–24] and SGA [25–27]. Therefore, it has been hypothesized that low antioxidant levels in pregnancy may increase the risk of preeclampsia and SGA.

One systematic review and meta-analysis of observational studies has assessed the association between lipid peroxidation and/or antioxidant status in pregnancy and preeclampsia [28]. That review showed that markers of lipid peroxidation were, on average, higher in preeclampsia cases, and vitamins C and E were significantly lower. A number of important limitations of this review justify further evaluation. Substantial heterogeneity was observed but not investigated. Many studies published at the time were not included or described, including at least one prospective study [29]. Further, the review did not include SGA as an outcome which is of interest as SGA and preeclampsia often appear together [8].

The findings of early observational studies and the success of a small pilot intervention study [30], inspired a number of large randomized controlled trials (RCTs) to assess the use of antioxidant supplements to prevent preeclampsia and associated sequelae, including SGA [31–34]. Disappointingly, these trials mostly showed null results. Our goal was to summarize studies that formed the empirical basis for trials, investigate sources of heterogeneity, and investigate whether observational studies suffered from specific shortcomings. We conducted a systematic review of studies of the maternal levels of non-enzymatic antioxidants that could be given as dietary supplements to pregnant women. Ultimately, this review will enable us to understand whether additional observational studies or trials are warranted to comprehensively assess the suitability of antioxidant supplements for prevention of preeclampsia and SGA.

## Methods

We followed MOOSE guidelines in planning and carrying out the systematic review and meta-analysis [35]. Before undertaking the review, we registered our systematic review protocol in the PROSPERO database (S1 Protocol).

We searched for studies published in English or French from 1970-January 2013 that measured maternal blood levels of non-enzymatic antioxidants in any population of pregnant women. We were interested in antioxidant micronutrients that are obtained from the diet and dietary supplements that are present in measurable quantities in blood, and cannot be synthesized by the body. Such antioxidants included tocopherols (vitamin E), carotenes (α-carotene, β-carotene, lycopene), oxy-carotenoids (lutein, zeaxanthin, cryptoxanthin), retinol (vitamin A), and ascorbic acid (vitamin C). We included studies in which samples were obtained during pregnancy or within 72 hours of delivery. Pregnancy outcomes of interest included any preeclampsia, mild preeclampsia, severe preeclampsia (including eclampsia), early- and late-onset preeclampsia, and SGA or intrauterine growth restriction (IUGR; defined by serial ultrasounds). We included prospective cohort studies, nested case-control studies, RCTs (analyzed as cohorts), case-control studies and cross-sectional studies in our search.

### Sources

We searched the following databases without language restrictions: Pubmed; Medline, Embase, CAB Abstracts, and BIOSIS Previews via OvidSP; ProQuest Dissertations & Theses, CINAHL, FSTA Direct, and POPLINE. The search strategy was developed in consultation with an experienced health sciences librarian. We pilot tested the ability of our search strategy to identify records of known studies and further refined it after preliminary searches to include all relevant controlled vocabulary (subject headings that included MeSH terms in Medline and Emtree terms in Embase) and free-text words in the title or abstract.

Search terms for exposures included: antioxidant, tocopherol, vitamin E, beta-carotene, carotene, carotenoid, lycopene, cryptoxanthin, lutein, vitamin A, retinol, vitamin C, ascorbic acid, and ascorbate. Search terms for outcomes were: preeclampsia, pre-eclampsia, toxemia, small for gestational age, SGA, fetal growth retardation, fetal growth restriction, FGR, intrauterine growth retardation, intrauterine growth restriction, and IUGR. The complete Pubmed search is shown in **Appendix A in** S1 File.

In addition to the above database searches, we also hand-searched the reference lists of relevant retrieved studies and of narrative and systematic reviews to find additional studies. Unpublished literature was sought by searching the ProQuest Dissertations & Theses database.

### Study Selection

Two trained reviewers independently screened all titles and abstracts of records retrieved from database searches. Records considered potentially relevant by one or both reviewers proceeded to the secondary screen of full-text articles. Two reviewers independently assessed each of these studies using a structured study eligibility form. Where there were disagreements, the reviewers discussed and came to a consensus, sought additional information from authors to make a decision, or discussed with a third reviewer.

We excluded studies that did not provide diagnostic criteria for the outcome of interest and those that did not provide gestational age (GA) at time of blood sampling. We were interested in whether differences in diagnostic criteria and timing of biomarker measurement could explain heterogeneity of results; hence we required that these be reported. We excluded studies that obtained samples >72 hours after delivery, as antioxidant levels obtained more than a few days after delivery may not be representative of levels during pregnancy [36]. We excluded studies published in a language other than English or French, owing to resource constraints that limited our ability to obtain translations. Finally, we excluded studies published prior to 1970, when high-performance liquid chromatography technology was developed used for precise measurement of many antioxidant biomarker levels [37, 38]. No review articles, letters to the editor, or duplicate publications were included; however, published abstracts were considered eligible. In the case of abstracts, definition of the outcome was not required due to acknowledged space constraints.

### Data Extraction

For studies deemed eligible for inclusion based on the secondary full-text screen, data were independently extracted by two reviewers using a data extraction form developed and pilot tested for this literature. The following data were extracted from each study:

- Study characteristics: study design, setting (country), time period
- Population characteristics: inclusion/exclusion criteria, covariates measured, prevalence of risk factors, by study group
- Description of cases and controls: definition of preeclampsia and/or SGA, gestational age distribution at recruitment (first blood draw), characteristics of study controls (and matching criteria, if applicable)
- Exposure characteristics: timing of antioxidant measurement, antioxidants assessed, biomarker assay methods, adjustment for blood lipids (yes/no, method)
- Outcome: mean and SD of each antioxidant measured, by group; measures of association for preeclampsia and SGA, covariates included in multivariable models

Where data for meta-analysis were available only in figures, we extracted the data using WebPlotDigitizer Version 3.4, a free online program (Rohatgi A. WebPlotDigitizer. 3.4 ed. http://arohatgi.info/WebPlotDigitizer; 2014).

Methodological quality and potential biases were also evaluated for each study. Data were entered into two separate Access 2010 databases (Microsoft, Redmond, WA) and compared for agreement. Disagreements were resolved by coming to consensus or discussing with a third reviewer.

### Risk of Bias Assessment

Two reviewers independently assessed the quality of each study using the Newcastle-Ottawa Scales (NOS) for cohort and case-control studies (http://www.ohri.ca/programs/clinical_epidemiology/oxford.asp). This scale uses multiple-choice questions to address the areas of selection, comparability, and exposure/outcome assessment. Points (stars) are given for high-quality rating in each of these areas and studies earned a maximum of 9 points. We tailored the NOS to this literature by specifying the key confounders *a priori*: gestational age, smoking, parity, and BMI. We also rated studies on overall quality of reporting and confounding control using “risk of bias” additional questions. We prepared a manual to guide our assessments and make the judgements as consistently as possible (**Appendix B in** S1 File).

### Statistical Analysis

We conducted meta-analyses for four distinct outcomes: all preeclampsia, mild preeclampsia, severe preeclampsia or eclampsia, and SGA. Since the antioxidants were reported in different units, we pooled the standardized mean difference (SMDs) across three or more studies for each antioxidant, stratified by trimester of measurement. The SMD provides an estimate of the average difference, in standard deviations, between cases and controls in the levels of a continuous exposure, when different units are reported across studies. We converted standard errors to standard deviations; where the median and range of values were reported, and we estimated the mean and SD using the method of Hozo *et al* [39]. We pooled subgroup means and standard deviations (e.g. combined mild and severe for all preeclampsia analysis) using formulae described in the Cochrane Handbook (details in **Appendix C in** S1 File) [40]. Meta-analysis was carried out using Dersimonian and Laird random effects models, with I^2^ used to quantify heterogeneity.

For our primary analysis of vitamin E status, we constructed a composite variable which refers to α-tocopherol status where available, or vitamin E provided as total tocopherol or unspecified. We conducted influence analyses to assess the robustness of our findings by assessing whether any individual studies were highly influential on the pooled result, sequentially excluding one study at a time from the analysis.

We investigated heterogeneity due to study design, quality, population, setting, exposure characteristics, variation in definition of severe preeclampsia, and possible confounding by gestational age, maternal age, and BMI via univariate and multivariate random-effects meta-regression. We classified study designs as prospective if they measured biomarker levels before preeclampsia or SGA became clinically apparent. Study quality was assessed by (1) whether authors addressed confounding in the design or analysis (yes or unclear versus no), (2) NOS score at or above versus below the median. We stratified by study population in three categories: general, high-risk only, or other. We stratified studies carried out exclusively in low- and middle-income countries (LMIC), where nutritional deficiencies may be more common, vs. high-income countries (HIC). For exposure characteristics, we assessed fasting status, assay method (high-performance liquid chromatography, HPLC vs. other), and timing of sample collection (before versus during or after labor). For vitamin E, we assessed whether the magnitude of association differed for total tocopherol or unspecified vs. α-tocopherol. Finally, we calculated the mean difference in gestational age, maternal age, and BMI between cases and controls in each study and used this variable in meta-regression analyses to assess potential confounding by these characteristics. As a sensitivity analysis for vitamin E studies, we restricted to studies measuring α-tocopherol to see if meta-regression findings were consistent.

Publication bias occurs when studies with null findings are less likely to be published than those finding a significant association [41]. As recommended by Sterne, Egger and Davey Smith, we examined publication bias by using funnel plots [41]. We also restricted to higher-quality studies (those that addressed confounding in design or analysis) to assess whether publication bias was less evident among them.

Stata 11 (StataCorp, College Station, Texas) was used for statistical analyses and for generating figures.

## Results

**Fig 1:**
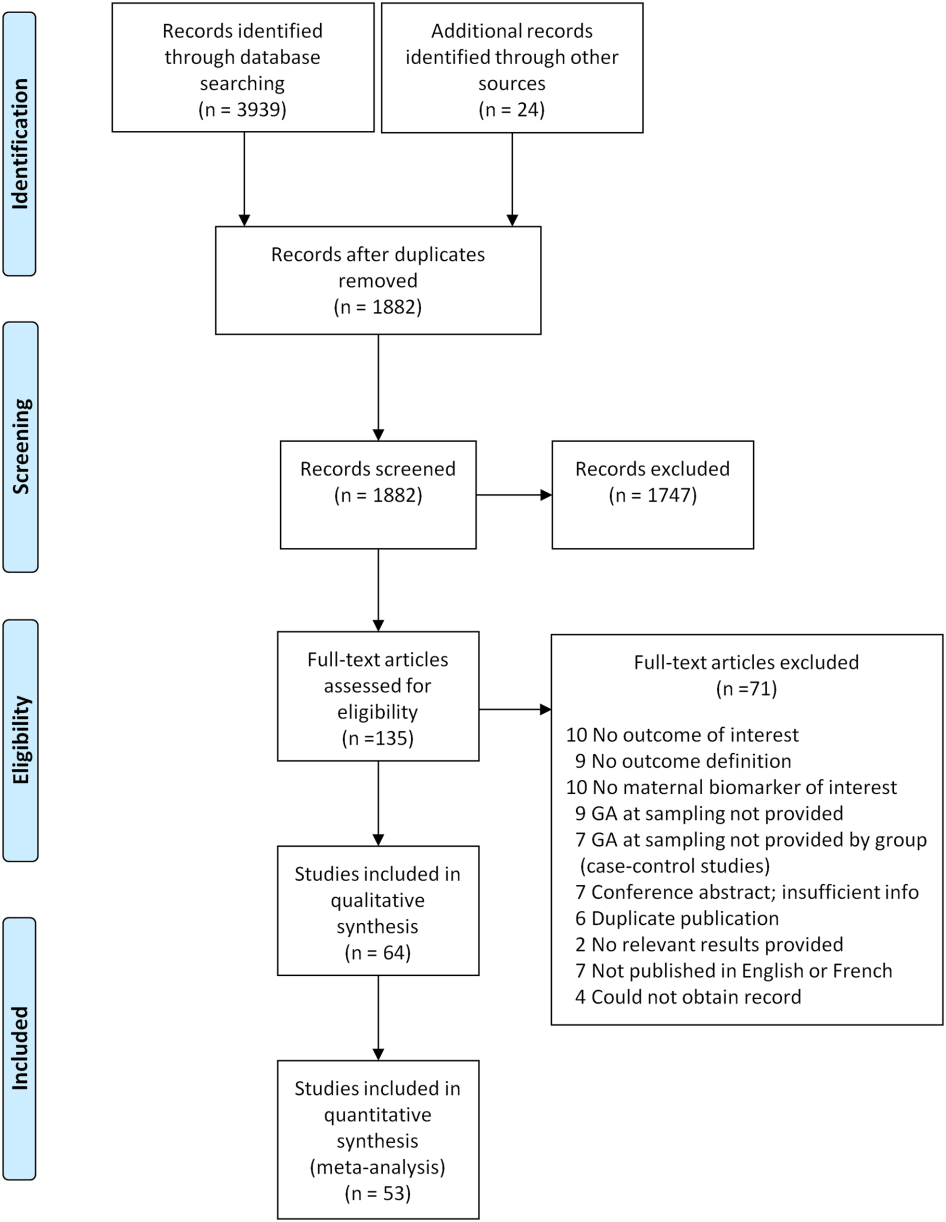
Flow diagram of study selection process. Figure adapted from: Moher D, Liberati A, Tetzlaff J, Altman DG, The PRISMA Group (2009). Preferred Reporting Items for Systematic Reviews and Meta-Analyses: The PRISMA Statement. PLoS Med 6(6): e1000097. doi:10.1371/journal.pmed1000097

Database searches returned 3,939 total records and search of reference lists of relevant studies and literature reviews identified an additional 24 records (Fig 1). Reviewers completed the title and abstract screen on 1,882 unique records and selected 135 for full-text review and eligibility assessment. Sixty-four studies published from 1973 to 2012 were selected for inclusion. The complete list of included studies is provided in **Appendix D in** S1 File. Fifty-five studies addressed the outcome preeclampsia, six addressed SGA, and three addressed both outcomes (Table 1). We were unable to identify any studies of antioxidant levels in relation to IUGR, defined by serial ultrasounds; however, some included studies defined IUGR as birthweight <10^th^ percentile and we referred to the outcome in these studies as SGA. Sample sizes ranged from 20 to 1231 subjects. Forty-two studies (66%) were conducted in general population samples; others were carried out in selected groups, including women with diabetes, HIV, high-risk for preeclampsia, first pregnancy, elective cesarean, etc. The most common antioxidant assessed was vitamin E (unspecified, total tocopherol, or α-tocopherol), followed by vitamins C and A.

**Table 1:** Characteristics of included studies.

Study	Design	Country	Population	Study Groups	Group Ns	NOS	RB7*
PREECLAMPSIA STUDIES							
Akyol et al. (2000)	Case-control	Turkey	General population	Controls / Mild / Severe PE	15 / 17 / 16	5	Unclear
Azar et al. (2011)	Nested case-control	Norway, Australia, USA	Women with diabetes	Controls / PE Cases	24 / 23	7.5	Yes
Bakheit et al. (2010)	Case-control	Sudan	General population	Controls / PE Cases	38 / 37	5	Yes
Basu and Arulanantham (1973)	Cross-sectional	India	Low SES women	Controls / PE Cases	56 / 50	1	No
Ben-Haroush et al. (2002)	Nested case-control	Israel	High-risk for PE	Controls / PE Cases	36 / 8	4	Unclear
Bowen et al. (1998)	Case-control	S. Africa	Low SES, black African	Controls / Mild / Severe PE	32 / 15 / 31	5	Unclear
Bowen et al. (2001)	Case-control	S. Africa	First pregnancy	Controls / PE / Eclampsia	29 / 21 / 6	3.5	No
Dehghan, Daryani, and Dehghanan (2007)	Case-control	Iran	General population	Controls / PE Cases	100 / 50	5.5	Unclear
Dirican et al. (2008)	Case-control	Turkey	General population	Term Controls / Mild / Severe PE	20 / 21 / 21	4	No
Elsen et al. (2012)	RCT sub-study	Venezuela, Tanzania	High-risk for PE	Controls / Mild / Severe PE	394 / 20 / 17	5	No
Gratacos et al. (1999)	Case-control	Spain	General population	Term Controls / PE / Superimposed	36 / 34 / 11	6	No
Harma and Erel (2005)	Case-control	Turkey	General population	Control / PE Cases	18 / 24	4.5	Unclear
Harsem, Braekke, and Staff (2006)	Case-control	Norway	Elective cesarean	Control / PE Cases	38 / 21	4	No
Harsem et al. (2008)	Case-control	Norway	Elective cesarean	Control / PE Cases	38 / 36	3	No
Howlader et al. (2007)	Case-control	Bangladesh	General population	Control / PE Cases	22 / 25	3.5	Unclear
Hubel et al. (1997)	Case-control	USA	General population	Control / PE Cases	17 / 14	4.5	No
Ikpen et al. (2012)	Case-control	Nigeria	General population	Control / PE Cases	80 / 80	7	Yes
Islam et al. (2004)	Case-control	Bangladesh	General population	Control / PE Cases / Eclampsia	35 / 44 / 50	4	Unclear
Jendryczko and Drozdz (1989)	Prospective cohort	NR (Poland)	General population	Term Controls / PE Cases	11 / 9	2	No
Kaur et al. (2008)	Case-control	India	General population	Controls / Mild / Severe PE	56 / 47 / 77	1	Unclear
Kharb (2000)	Case-control	India	First pregnancy	Controls / PE Cases	30 / 30	5	Yes
Kiondo et al. (2012)	Case-control	Uganda	General population	Controls / PE Cases	400 / 215	6.5	Unclear
Kolusari et al. (2008)	Case-control	Turkey	General population	Controls / PE Cases	48 / 47	4	No
Llurba et al. (2004)	Case-control	Spain	General population	Controls / PE Cases	30 / 53	5.5	Yes
Madazli et al. (1999)	Case-control	Turkey	General population	Controls / PE Cases	21 / 22	4	No
Mehendale et al. (2008)	Case-control	India	General population	Controls / PE Cases	55 / 60	4.5	Unclear
Mikhail et al. (1994)	Case-control	USA	General population	Controls / Mild / Severe PE	44 / 22 / 8	5	No
Mohanty et al. (2006)	Case-control	India	First pregnancy, low SES	Controls / Mild / Severe PE	20 / 38 / 12	3.5	Unclear
Mohindra et al. (2002)	Case-control	India	Primiparous women	Controls / PE Cases	54 / 33	2.5	No
Morris et al. (1998)	Case-control	UK	General population	Controls / PE Cases	19 / 19	4	Yes
Mutlu-Turkoglu et al. (1998)	Case-control	Turkey	General population	Controls / PE Cases	10 / 17	4.5	Unclear
Nilar et al. (2009)	Case-control	Myanmar	General population	Controls / PE Cases	48 / 25	5	No
Noyan et al. (2006)	Case-control	Turkey	General population	Controls / PE / Eclampsia	19 / 21 / 11	3	No
Ozan et al. (1997)	Case-control	Turkey	General population	Controls / Mild / Severe PE / Eclampsia	20 / 14 / 12 / 13	4	No
Palan, Mikhail, and Romney (2001)	Case-control	USA	General population	Controls / PE Cases	22 / 19	5	Yes
Palan et al. (2004)	Case-control	USA	General population	Controls / Mild / Severe PE	42 / 25 / 28	5	No
Panburana, Phuapradit, and Puchaiwatananon (2000)	Case-control	Thailand	General population	Controls / Mild / Severe PE	60 / 30 / 20	3	No
Roland et al. (2010)	Case-control	Canada	General population	Controls / PE Cases	30 / 29	3	Unclear
Sagol, Ozkinay, and Ozsener (1999)	Case-control	Turkey	General population	Term Controls / Mild / Severe PE	33 / 8 / 16	5.5	Unclear
Serdar et al. (2003)	Case-control	Turkey	Elective cesarean	Term Controls / Mild / Severe PE	50 / 30 / 30	4	No
Serdar et al. (2002)	Case-control	Turkey	Elective cesarean	Term Controls / PE Cases	72 / 70	3	No
Sharma et al. (2006)	Case-control	India	General population	Controls / PE Cases	50 / 50	2.5	Unclear
Sharma et al. (1984)	Case-control	NR	General population	Controls / Moderate / Severe PE	86 / 12 / 41	3	No
Suhail and Faizul-Suhail (2009)	Case-control	India	General population	Controls / Severe PE Cases	21 / 21	4	No
Uotila et al. (1994)	Case-control	Finland	Elective cesarean	Controls / PE Cases	9 / 11	4	No
Uotila et al. (1993)	Case-control	Finland	General population	Controls / Mild / Severe PE	20 / 20 / 23	3	No
Wei et al. (2012)	Nested case-control	Canada, Mexico	High-risk & low-risk RCT participants (INTAPP trial)	Controls / PE Cases	229 / 115	5.5	Yes
Wikstrom et al. (2009)	Case-control	Sweden	General population	Early Controls / Early PE / Late Controls / Late PE	22 / 18 / 18 / 20	3.5	Unclear
Williams et al. (2003)	Case-control	Zimbabwe	General population	Controls / PE	186 / 173	7.5	Yes
Xu (2011)	Nested case-control	Canada, Mexico	High-risk & low-risk RCT participants (INTAPP trial)	Controls / PE Cases	229 / 115	7.5	Yes
Yanik et al. (1999)	Case-control	Turkey	General population	Controls / PE / Eclampsia	25 / 18 / 15	5	No
Zhang, Luthy, et al. (2001)	Nested case-control	NR	General population	Controls / PE Cases	241 / 24	7	Yes
Zhang, Williams, et al. (2001)	Case-control	Peru	General population	Controls / PE Cases	179 / 125	7.5	Yes
Ziari et al. (1996)	Case-control	Nigeria	1^st^ or 2^nd^ pregnancy	Controls / PE / Eclampsia	16 / 9 / 7	4	Unclear
Zusterzeel et al. (2002)	Case-control	Netherlands	General population	Controls / Mild PE	14 / 14	5	No
PREECLAMPSIA & SGA STUDIES							
Chappell et al. (2002)	Semi-nested case-control	UK	High-risk cases; low-risk controls	Controls / PE / SGA Cases	27 / 21 / 17	5.5	Unclear
Rajasingam et al. (2009)	RCT sub-study	UK, Netherlands	High-risk for PE (obese, nulliparous)	Total Cohort	385	7	Unclear
Schiff et al. (1996)	Case-control	USA	General population	Controls / PE Cases / SGA+PE (subgroup of PE)	90 / 48 / 14	5	No
SGA STUDIES							
Agarwal et al. (2008)	Cross-sectional	India	Healthy term mother-newborn pairs	Control / SGA Cases	30 / 30	4	No
Dreyfuss et al. (2001)	RCT sub-study	Tanzania	HIV-positive women	Total Cohort	822	6	Yes
Kerver et al. (2012)	Nested case-control	USA	General population (POUCH study)	Total Cohort	547	4	Unclear
Ortega-Senovilla et al. (2010)	Case-control	Italy	General population	Controls / SGA Cases	25 / 24	5	Unclear
Saker et al. (2008)	Prospective cohort	Algeria	Term births	Controls / SGA Cases	56 / 45	6	Unclear
Scholl et al. (2006)	Prospective cohort	USA	General population (Camden study)	Total Cohort	1231	6.5	Yes

*RB7, Risk of Bias question #7 (“Was confounding accounted for in the design and/or analysis?”); PE, preeclampsia; SGA, small for gestational age; NOS, Newcastle-Ottawa Scale [score out of 9 maximum]; SES, socioeconomic status; INTAPP, An international trial of antioxidants in the prevention of preeclampsia, POUCH, Pregnancy outcomes and community health

The 58 preeclampsia studies comprised 48 traditional case-control studies, 1 cross-sectional study, and 9 prospective designs (nested case-control, cohort, RCT). Thirty-three were conducted exclusively in LMICs; most commonly Turkey (n = 11) and India (n = 8). Fourteen studies stratified cases as mild and severe for their primary analysis. Only one stratified early- (<34 weeks) and late-onset preeclampsia. Biomarker measurement spanned all three trimesters across the reviewed literature (Table 2). Three studies reported serial measures of biomarker levels.

**Table 2:** Details of antioxidant biomarker measurements.

Study	Outcome	Markers measured	Sample Timing	GA Range (wk)	Fasting	Units
Akyol et al. (2000)	PE	Vitamin E**	Before delivery	28–40	Unclear	mg/dL
Azar et al. (2011)	PE	Retinol, α-, γ-tocopherol, α-, β-carotene, lycopene, lutein	3 samples; once per trimester		Yes	umol/L
Bakheit et al. (2010)	PE	Retinol, α-tocopherol	Before delivery	Third trimester	Unclear	ug/dL
Basu and Arulanantham (1973)	PE	Retinol*	Before delivery	25–40	Unclear	ug/dL
Ben-Haroush et al. (2002)	PE	Vitamin E*	Before labor	7–32	Yes	mg/dL
Bowen et al. (1998)	PE	Vitamin C*, vitamin E	Before labor	Third trimester	Unclear	umol/L
Bowen et al. (2001)	PE	Vitamin C, vitamin E	Before delivery	Third trimester	Unclear	umol/L
Dehghan, Daryani, and Dehghanan (2007)	PE	Vitamin C*	Before delivery	28–42	Unclear	mg/dL
Dirican et al. (2008)	PE	Vitamin C*, vitamin E*, total carotene*	Before delivery	Third trimester	Unclear	umol/L
Elsen et al. (2012)	PE	Retinol, vitamin E	7 samples; every 4 weeks from week 12		Unclear	ug/L
Gratacos et al. (1999)	PE	α-tocopherol	Before labor	Third trimester	Yes	mg/mL
Harma and Erel (2005)	PE	Vitamin C*	Before labor	Third trimester	Unclear	umol/L
Harsem, Braekke, and Staff (2006)	PE	α-tocopherol	Before labor	25–40	Yes	umol/L
Harsem et al. (2008)	PE	Vitamin E	Before labor	24–40	Yes	umol/L
Howlader et al. (2007)	PE	Vitamin C*	Before delivery	Third trimester	Unclear	mg/dL
Hubel et al. (1997)	PE	Vitamin C*, α-tocopherol	Before delivery	Third trimester	Unclear	nmol/mL
Ikpen et al. (2012)	PE	Vitamin C*, vitamin E*	Before delivery	Third trimester	Unclear	mg/dL
Islam et al. (2004)	PE	Retinol, vitamin C*, α-tocopherol	Before delivery	28–42	Unclear	umol/L
Jendryczko and Drozdz (1989)	PE	Retinol, vitamin E, β-carotene	5 samples; Every 2 weeks from week 28		Unclear	ug/L
Kaur et al. (2008)	PE	Retinol, vitamin C*, vitamin E	Before delivery	Third trimester	Unclear	ug/mL, mg% (vitamin C)
Kharb (2000)	PE	Vitamin C*, Vitamin E*	Before labor	28–40	Yes	umol/L
Kiondo et al. (2012)	PE	Vitamin C*	Before delivery	20-	Unclear	mg/L
Kolusari et al. (2008)	PE	Retinol, α-tocopherol	Before delivery	29–38	Yes	mmol/L
Llurba et al. (2004)	PE	Vitamin C, α-tocopherol	Before labor	24–40	Yes	umol/L
Madazli et al. (1999)	PE	Vitamin C*, α-tocopherol*	Before delivery	Third trimester	Yes	mg/dL
Mehendale et al. (2008)	PE	Vitamin C*, α-tocopherol*	Before labor	36-	Unclear	mg%
Mikhail et al. (1994)	PE	Retinol, α-tocopherol, β-carotene	Before labor	28–42	Yes	mg/dL
Mohanty et al. (2006)	PE	Vitamin C*, vitamin E*	Before delivery	28–42	Yes	mg/L, mol/L (vitamin E)
Mohindra et al. (2002)	PE	Total carotene (NR)	Before delivery	Third trimester	Yes	ug/dL
Morris et al. (1998)	PE	Vitamin E	Before delivery	Third trimester	Unclear	ug/mL
Mutlu-Turkoglu et al. (1998)	PE	Vitamin C*	Before labor or after delivery	Third trimester	Unclear	umol/L
Nilar et al. (2009)	PE	Vitamin C*, vitamin E*	Before labor	28–40	Unclear	mg/dL
Noyan et al. (2006)	PE	Vitamin C*	Before delivery	23–40	Yes	mg/dL
Ozan et al. (1997)	PE	Vitamin C*	Before delivery	Third trimester	Yes	mg/dL
Palan, Mikhail, and Romney (2001)	PE	α-, β-carotene, lycopene, canthaxanthin	After delivery	30–42	Unclear	ug/dL
Palan et al. (2004)	PE	α-, γ-tocopherol	Before delivery	30–41	No	umol/L
Panburana, Phuapradit, and Puchaiwatananon (2000)	PE	Retinol, vitamin C*, α-tocopherol	Before delivery	Third trimester	Yes	umol/L
Roland et al. (2010)	PE	α-, γ-tocopherol, total tocopherol, β-carotene	Before delivery	Third trimester	Unclear	umol/L
Sagol, Ozkinay, and Ozsener (1999)	PE	Vitamin C, α-tocopherol	Before delivery	20–40	Unclear	ug/L
Serdar et al. (2003)	PE	Vitamin E*, total carotene*	Before labor	31–38	Yes	umol/L
Serdar et al. (2002)	PE	Vitamin E*, total carotene*	Before labor	31–38	Yes	umol/L
Sharma et al. (2006)	PE	Vitamin C*, lycopene	Before delivery	26–42	Unclear	mg/dL
Sharma et al. (1984)	PE	Vitamin C*	Before delivery	28–40	Unclear	mg/dL
Suhail and Faizul-Suhail (2009)	PE	Retinol*, vitamin C*, vitamin E*	Before delivery	Third trimester	Unclear	umol/L
Uotila et al. (1994)	PE	Vitamin C, vitamin E	Before labor	Third trimester	Yes	umol/L
Uotila et al. (1993)	PE	Vitamin E	Before delivery	31–38	Unclear	umol/L
Wei et al. (2012)	PE	β-carotene	3 samples; 12–18 (pre-intervention), 24–26, 32–34 weeks		Unclear	ug/mL
Wikstrom et al. (2009)	PE	Vitamin C*, α-tocopherol	Before delivery	Third trimester	Unclear	umol/L
Williams et al. (2003)	PE	Retinol, α-, γ-tocopherol, α-, β-carotene, lycopene, lutein, zeaxanthin, β-cryptoxanthin	After delivery (12–72 hours)	Third trimester	Unclear	ug/mL
Xu (2011)	PE	α-, γ-tocopherol, total tocopherol	3 samples; 12–18 (pre-intervention), 24–26, 32–34 weeks		Unclear	NR
Yanik et al. (1999)	PE	Vitamin E*	Before delivery	28–41	Unclear	mg/mL
Zhang, Luthy, et al. (2001)	PE	Vitamin C	Before labor	First trimester	Unclear	umol/L
Zhang, Williams, et al. (2001)	PE	Retinol, α-, γ-tocopherol, α-, β-carotene, lycopene, lutein, zeaxanthin, β-cryptoxanthin	Before delivery	Third trimester	Unclear	umol/L
Ziari et al. (1996)	PE	Retinol, α-tocopherol, β-carotene	Before labor	Third trimester	Yes	ug/dL
Zusterzeel et al. (2002)	PE	Vitamin C, α-tocopherol	Before labor	Third trimester	Yes	umol/L
Chappell et al. (2002)	PE & SGA	Vitamin C, α-tocopherol	5 samples; every 4 weeks from week 20		Unclear	umol/L
Rajasingam et al. (2009)	PE & SGA	Retinol, vitamin C, α-, γ-tocopherol	Before labor	14–22	No	umol/L
Schiff et al. (1996)	PE & SGA	Vitamin E	Before delivery	22–41	Unclear	mg/dL
Agarwal et al. (2008)	SGA	Retinol*	After delivery	37–42	Unclear	ug/dL
Dreyfuss et al. (2001)	SGA	Retinol, vitamin E (NR)	Before labor	12–27	Unclear	umol/L
Kerver et al. (2012)	SGA	Retinol, α-, γ-tocopherol, total carotenoids	Before labor	16–27	Unclear	not reported
Ortega-Senovilla et al. (2010)	SGA	Retinol, α-, γ-tocopherol	Before labor	Third trimester	Yes	umol/L
Saker et al. (2008)	SGA	Retinol, vitamin C*, α-tocopherol	After delivery	37–42	Yes	umol/L
Scholl et al. (2006)	SGA	α-, γ-tocopherol	2 samples	16 & 28 weeks	Unclear	ug/mL

NR, not reported; PE, preeclampsia; SGA, small for gestational age;

* = Spectrophotometric method,

** = Enzymatic Assay (method is HPLC unless otherwise indicated)

Among the 9 SGA studies, 2 were case-control studies, 1 was cross-sectional, and 6 used prospective designs. Three studies were conducted in LMICs. These studies measured biomarker levels in the 2^nd^ and 3^rd^ trimesters; hence there were no SGA studies reporting first-trimester levels of any of the exposures of interest. Two studies reported serial measures of biomarker levels.

### Risk of Bias

The results of the risk of bias assessment are shown in Table 3. With “No” being the rating associated with the highest risk of bias, the studies were weakest with respect to accounting for confounding in the design or analysis. Twenty-eight studies (44%) did not account for confounding and another 22 (34%) were rated as unclear because of a concern of important residual confounding.

**Table 3:** Study quality items assessed in addition to Newcastle-Ottawa Scale.

Quality Assessment Item	Yes—n (%)	Unclear—n (%)	No—n (%)
RB1. Was the study clinical setting well-described?	28 (44)	25 (39)	11 (17)
RB2. Were incomplete data (i.e. missing data) adequately described?	13 (20)	47 (73)	4 (6)
RB3. Were statistical analyses described adequately?	41 (64)	15 (23)	8 (13)
RB4. Were analyses appropriate?	39 (61)	24 (38)	1 (2)
RB5. Did analysis provide sufficient presentation of data?	52 (81)	2 (3)	10 (16)
RB6. Is the study report free of the suggestion of selective reporting?	55 (86)	5 (8)	4 (6)
RB7. Was confounding accounted for in the design and/or analysis?	14 (22)	22 (34)	28 (44)

RB, “Risk of Bias”; Manual for assigning “yes”, “unclear,” and “no” available in Appendix B in S1 File. Percents may not sum to exactly 100 due to rounding

The median score for the Newcastle-Ottawa Scale was 4.5/9 (shown in Table 1) which is sub-optimal. Although there is no established cut-off for high-quality, several studies have used 6/9 to denote moderate or high-quality [42, 43]. Representativeness of cases was a concern in many of the case-control studies. Only 23% reported recruiting consecutive eligible cases or an otherwise obviously representative sample. Confounding was also an issue identified with the Newcastle-Ottawa scale. Thirty-eight percent of studies controlled for confounding by gestational age, 25% controlled for parity, 16% for smoking, and 11% for pre-pregnancy BMI.

### Data for Meta-Analysis

We included 53 studies in the quantitative synthesis of results. We extracted the mean and standard deviation or standard error from the figures of five studies [29, 44–47]. Descriptions of the data which prevented statistical pooling are detailed in **Appendix E in** S1 File. We were unable to pool ORs across studies since ORs were reported for various heterogeneous comparisons: across quantiles (tertiles, quartiles, quintiles), above or below a specified cut-off value or percentile, and per unit SD (z-score). Nine studies provided ORs for an association between one of the antioxidant biomarkers of interest and preeclampsia and/or SGA. Eight of these studies provided adjusted ORs.

### Vitamin E

**Fig 2:**
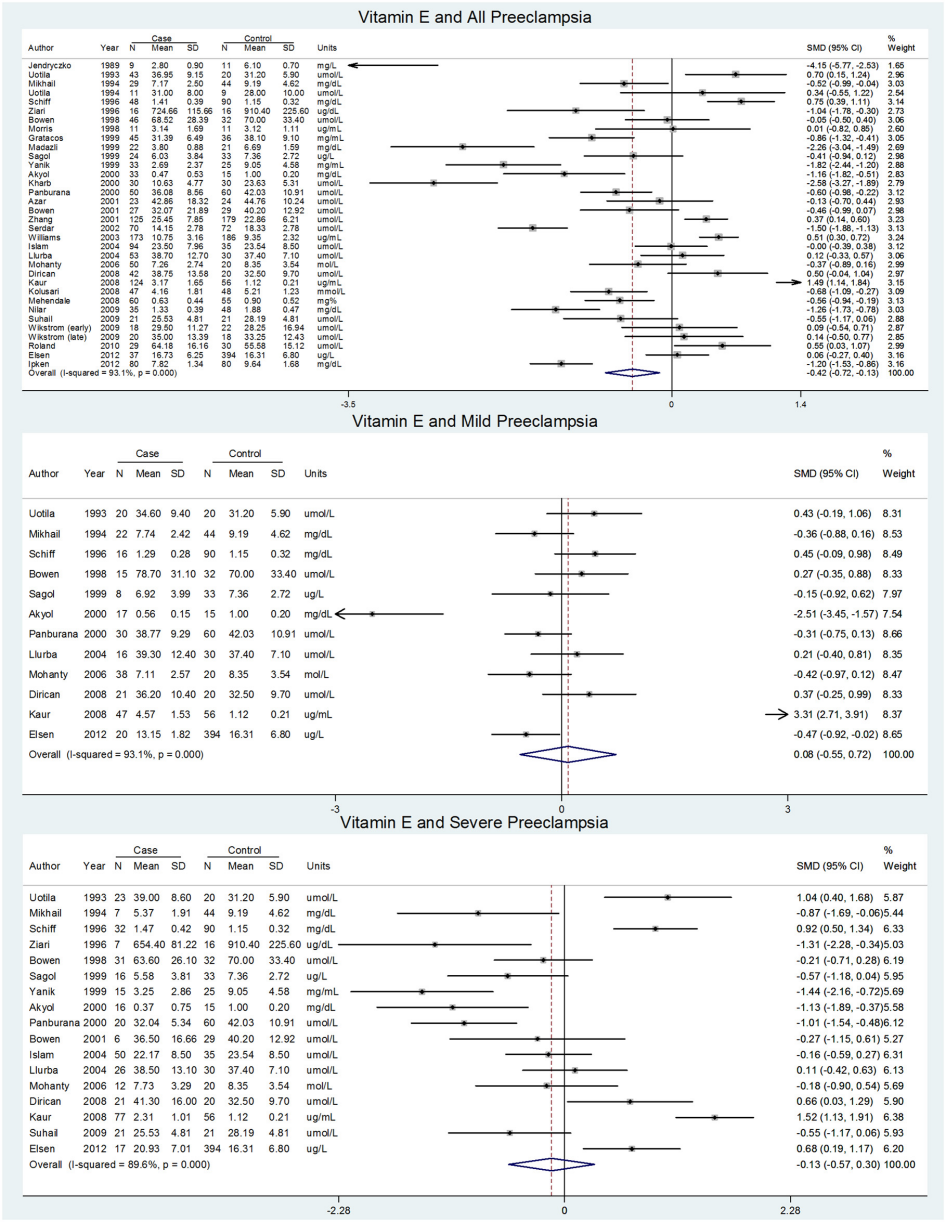
Forest plots for vitamin E and preeclampsia containing raw data.

Vitamin E reported as α-tocopherol, total tocopherol, or unspecified was measured in the first trimester in one study, second trimester in five studies, and third trimester in 41 studies in relation to preeclampsia. Meta-analysis of three studies estimated no difference in second trimester vitamin E levels between preeclampsia cases and controls; pooled SMD = 0.32 (-0.12, 0.76), I^2^ = 56% (0, 88%). The meta-analysis of 34 third trimester studies showed a significantly negative SMD but substantial heterogeneity (Table 4, Fig 2). For both mild and severe preeclampsia, however, the pooled SMDs were null with substantial heterogeneity (Table 4, Fig 2). Additional studies we were unable to pool had mixed results, consistent with the meta-analyses (**Appendix F in** S1 File).

**Table 4:** Meta-analyses of third trimester antioxidant levels.

		Random Effects Model			Heterogeneity	
Marker, Outcome	No. of Studies	SMD	95% CI	p-value	I^2^, %	95% CI
Retinol						
SGA	3	0.47	-0.56, 1.49	0.37	92	79, 97
All preeclampsia	12	-0.51	-0.88, -0.15	0.01	91	85, 94
Mild preeclampsia	3	-0.08	-0.58, 0.42	0.76	76	21, 93
Severe preeclampsia	6	-0.62	-1.58, 0.34	0.21	95	91, 97
Vitamin C						
All preeclampsia	29*	-0.56	-0.83, -0.28	<0.01	91	88, 93
Mild preeclampsia	11	-0.32	-0.67, 0.03	0.07	77	59, 87
Severe preeclampsia	15	-0.35	-0.72, 0.01	0.06	84	74, 90
Vitamin E						
All preeclampsia	34*	-0.42	-0.72, -0.13	0.01	93	91, 94
Mild preeclampsia	12	0.08	-0.55, 0.72	0.79	93	90, 95
Severe preeclampsia	17	-0.13	-0.57, 0.30	0.55	90	85, 93
Lipid-adj. Vitamin E						
All preeclampsia	10*	-0.04	-0.41, 0.33	0.82	86	76, 92
Mild preeclampsia	4	0.16	-0.48, 0.80	0.63	83	56, 93
Severe preeclampsia	5	-0.30	-1.17, 0.58	0.51	91	82, 95
α-tocopherol						
All preeclampsia	15*	-0.35	-0.66, -0.03	0.03	88	83, 92
Mild preeclampsia	4	-0.20	-0.47, 0.07	0.15	0	0, 85
Severe preeclampsia	6	-0.56	-0.99, -0.14	0.01	66	17, 86
Lipid-adj. α-tocopherol						
All preeclampsia	7*	0.02	-0.37, 0.41	0.91	82	65, 91
Others—All preeclampsia						
α-carotene	3	-0.01	-0.16, 0.15	0.94	0	0, 90
β-carotene	7	-0.40	-0.72, -0.08	0.01	74	45, 88
Total carotene	4	-1.06	-1.65, -0.47	<0.01	86	66, 94
Lycopene	4	-1.05	-2.09, -0.00	0.05	97	96, 99
Lutein	3	-0.07	-0.34, 0.20	0.61	61	0, 89

*Wikstrom *et al*. (2009) early-onset cases and controls and late-onset cases and controls were treated as two separate studies.

SGA, small for gestational age; SMD, standardized mean difference; CI, confidence interval

Many of the studies corrected each individual’s vitamin E for a measure of their blood lipid concentration (total cholesterol, total lipid, or other). None of the studies reported any significant difference in lipid-corrected vitamin E in the second trimester. Meta-analyses of third trimester levels did not suggest systematic differences between preeclampsia cases and controls for vitamin E, once differences in blood lipid levels were accounted for (Table 4). Four additional studies had consistent results (**Appendix F in** S1 File).

Vitamin E in the form of γ-tocopherol was measured in the first trimester in one study, in the second trimester in three studies, and in the third trimester in five studies. Most studies found no significant difference but results suggested that higher levels may be associated with a modest increase in risk of preeclampsia. We were unable to perform meta-analyses. Additional details on the results of individual studies in **Appendix F in** S1 File. ORs for vitamin E and preeclampsia presented in the reviewed studies are detailed in **Appendix G in** S1 File.

In a total of eight studies, vitamin E (reported as α-tocopherol, total tocopherol, or unspecified) was measured in the second trimester in five studies, and third trimester in five studies in relation to SGA. No meta-analyses were undertaken. Two studies with serial measures across both trimesters showed that lipid-adjusted α-tocopherol was not significantly different during the second trimester but became significantly lower in SGA cases versus controls at the start of the third trimester (28 weeks) [29, 48]. Additional details in **Appendix F in** S1 File.

All three studies that measured γ-tocopherol in the second trimester adjusted for total cholesterol, and each reported no difference between SGA cases and controls [48–50]. Two studies measured γ-tocopherol levels in the third trimester. One reported non-significantly higher γ-tocopherol in late third trimester [51] and the other study found no difference in γ-tocopherol adjusted for total cholesterol at 28 weeks [48]. Scholl *et al*. took serial measures at 16 and 28 weeks and reported no difference at either time point [48].

### Vitamin C

**Fig 3:**
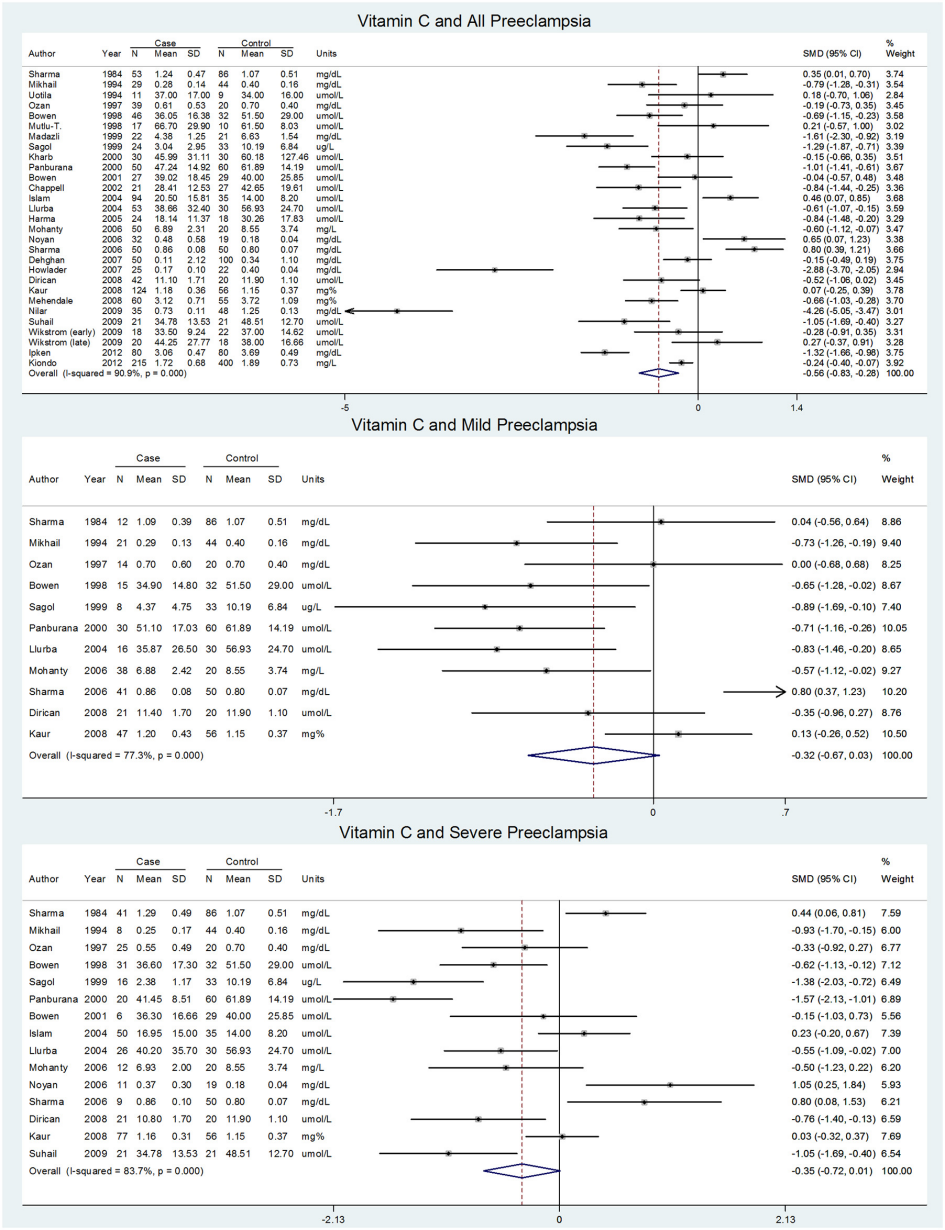
Forest plots for vitamin C and preeclampsia containing raw data.

Vitamin C was measured in the first trimester in one study, second trimester in two studies, and third trimester in 30 studies in relation to preeclampsia. The meta-analysis of 29 third trimester studies showed a significantly negative SMD but substantial heterogeneity (Table 4, Fig 3). For both mild and severe preeclampsia, the pooled SMDs were not significant with substantial heterogeneity (Table 4, Fig 3); however, the influence analysis revealed that exclusion of just one study in either case would have resulted in a significantly negative pooled SMD (**Appendix H in** S1 File). Additional studies we were unable to pool had mixed results, consistent with the meta-analyses (**Appendix F in** S1 File).

Vitamin C was measured in the second trimester in two studies, and third trimester in two studies in relation to SGA. The results showed consistently lower vitamin C levels in SGA cases compared to controls.

### Retinol/Vitamin A

Retinol was measured in the first trimester in one study, second trimester in two studies, and third trimester in thirteen studies in relation to preeclampsia. The meta-analysis of twelve third trimester studies showed a significantly negative SMD but substantial heterogeneity (Table 4). One additional study reported that retinol was non-significantly lower for preeclampsia cases versus controls [52]. For mild and severe preeclampsia where there were fewer studies, the pooled SMDs were null with substantial heterogeneity (Table 4).

Three studies found that retinol levels measured in the second trimester were similar among pregnancies resulting in SGA versus appropriate for gestational age (AGA) birth [49, 50, 53]. Three other studies measured retinol in the third trimester, all of which provided raw data for meta-analysis. Results were very heterogeneous (Table 4). Only one study measured levels before delivery and found significantly higher retinol in mothers who delivered SGA babies [51]. Two of the studies measured retinol levels shortly after delivery and found no significant differences for mothers who delivered SGA compared to AGA babies [27, 54].

### Carotenoids

Carotenes assessed in relation to preeclampsia included total carotene in five studies [21, 55–58], β-carotene in nine studies [22–24, 47, 59–63], α-carotene in four studies [24, 47, 60, 61], and lycopene in five studies [24, 47, 60, 61, 64]. Our meta-analyses estimated significantly negative pooled SMDs for total carotene, β-carotene, and lycopene with substantial heterogeneity (Table 4). For α-carotene, the SMD was not significant and the I^2^ was 0%.

Other carotenoids assessed in relation to preeclampsia in the reviewed studies included lutein [24, 47, 61], zeaxanthin [24, 61], β-cryptoxanthin [24, 61], and canthaxanthin [60]. Meta-analysis of three studies of lutein levels showed no significant difference between cases and controls (Table 4). Two large case-control studies did not report any ORs significantly different from 1 but subjects in the lowest quartiles of lutein, zeaxanthin, and β-cryptoxanthin had the highest odds of preeclampsia [24, 61]. Palan *et al*. reported that levels of canthazanthin were non-significantly lower in preeclampsia cases in the third trimester [60].

Kerver *et al*. reported an adjusted OR for SGA of 0.2 (95% CI 0.1, 0.8) for subjects above the 75^th^ percentile for total carotenoids measured in the second trimester [50]. It was unclear what factors were adjusted for since the study was a published abstract with limited information.

### Investigation of Heterogeneity

There were sufficient numbers of studies for a thorough investigation of heterogeneity among studies of vitamin E and C and preeclampsia. For Vitamin E and preeclampsia studies, we examined each factor in univariate models (Table 5). Since there were only three prospective studies in the meta-analysis, we restricted the multivariable model to studies measuring biomarker levels after diagnosis. Each factor that explained >1% of the variation, according to the adjusted R^2^, was included in the initial multivariable model. The final model described in the table included factors with p<0.1. Collectively, these factors explained some of the variation but the residual I^2^ remained above the threshold of 50% that is considered moderate heterogeneity. The model suggested that the negative SMD was driven by studies that used spectrometric methods as opposed to HPLC. More strongly negative SMDs were also observed among studies in which subjects were fasting. Among the studies of α-tocopherol, associations with HPLC and fasting were similar, and each explained some variation in the pooled SMD. A more negative SMD for studies with fasting samples and a less negative SMD for studies with HPLC measurement were also identified in the meta-regressions for mild and severe preeclampsia. However, univariable and multivariable models did not explain a large proportion of the variation amongst studies included in these models (**Appendix I in** S1 File).

**Table 5:** Meta-regression for vitamin E and any preeclampsia.

Covariate	No.	β-coef.	95% CI	P-value	I^2^, %	Adj. R^2^, %
Univariate models						
Prospective design	3	-0.60	-1.92, 0.73	0.37	93.3	-3.85
Study Quality						
Addressed Confounding	18	0.46	-0.24, 1.16	0.19	92.6	2.24
NOS>4.5 (above median)	15	-0.16	-0.88, 0.56	0.65	93.3	-2.71
Matched	9	0.31	-0.50, 1.11	0.45	93.1	-1.84
Population / Setting						
General Population	25	0.27	-0.54, 1.09	0.50	92.8	-1.12
High-risk for preeclampsia	6	-0.36	-1.29, 0.58	0.45	93.2	-1.07
LMIC	21	-0.46	-1.18, 0.27	0.21	93.2	4.51
Exposure Characteristics						
Fasting	12	-0.63	-1.34, 0.09	0.09	91.6	10.96
HPLC	23	1.07	0.42, 1.72	<0.01	88.6	35.71
Pre-labor sample	14	-0.57	-1.27, 0.13	0.11	92.2	5.77
α-tocopherol	16	0.23	-0.49, 0.94	0.53	93.1	-2.80
MD gestational age (per wk)	29	-0.09	-0.31, 0.13	0.41	92.8	-2.08
MD maternal age (per year)	28	0.00	-0.22, 0.23	0.96	91.0	-4.36
MD BMI (per unit kg/m2)	10	0.07	-0.24, 0.39	0.62	91.1	-9.04
Multivariable model intercept*	31	-0.85	-1.27, -0.43	<0.01	58.6	17.76
HPLC		1.15	0.67, 1.63	<0.01		
Fasting		-0.78	-1.26, -0.29	<0.01		

*Restricted to prospective studies. CI, confidence interval; HPLC, high-performance liquid chromatography; LMIC, low- or middle-income country; MD, mean difference; NOS, Newcastle-Ottawa Scale [score]

N overall = 34; SMD = -0.42, 95% CI -0.72, -0.13, I^2^ = 93%

For vitamin C and all preeclampsia, we were unable to explain heterogeneity in univariable meta-regressions. Although the studies by Howlader *et al* [45] and Nilar *et al* [65] were somewhat influential, based on the influence analysis, exclusion of neither study resulted in a change in the overall conclusion (**Appendix H in** S1 File).

For vitamin C and mild and severe preeclampsia, less heterogeneity was observed than for any preeclampsia. Matched studies had a pooled SMD closer to the null for severe preeclampsia. Whether or not the definition of severe PE included consideration of adverse conditions was important; for the definitions that more strictly relied on presence of severe hypertension and proteinuria, the pooled SMD was not significantly different from zero (**Appendix I in** S1 File).

### Publication Bias

**Fig 4:**
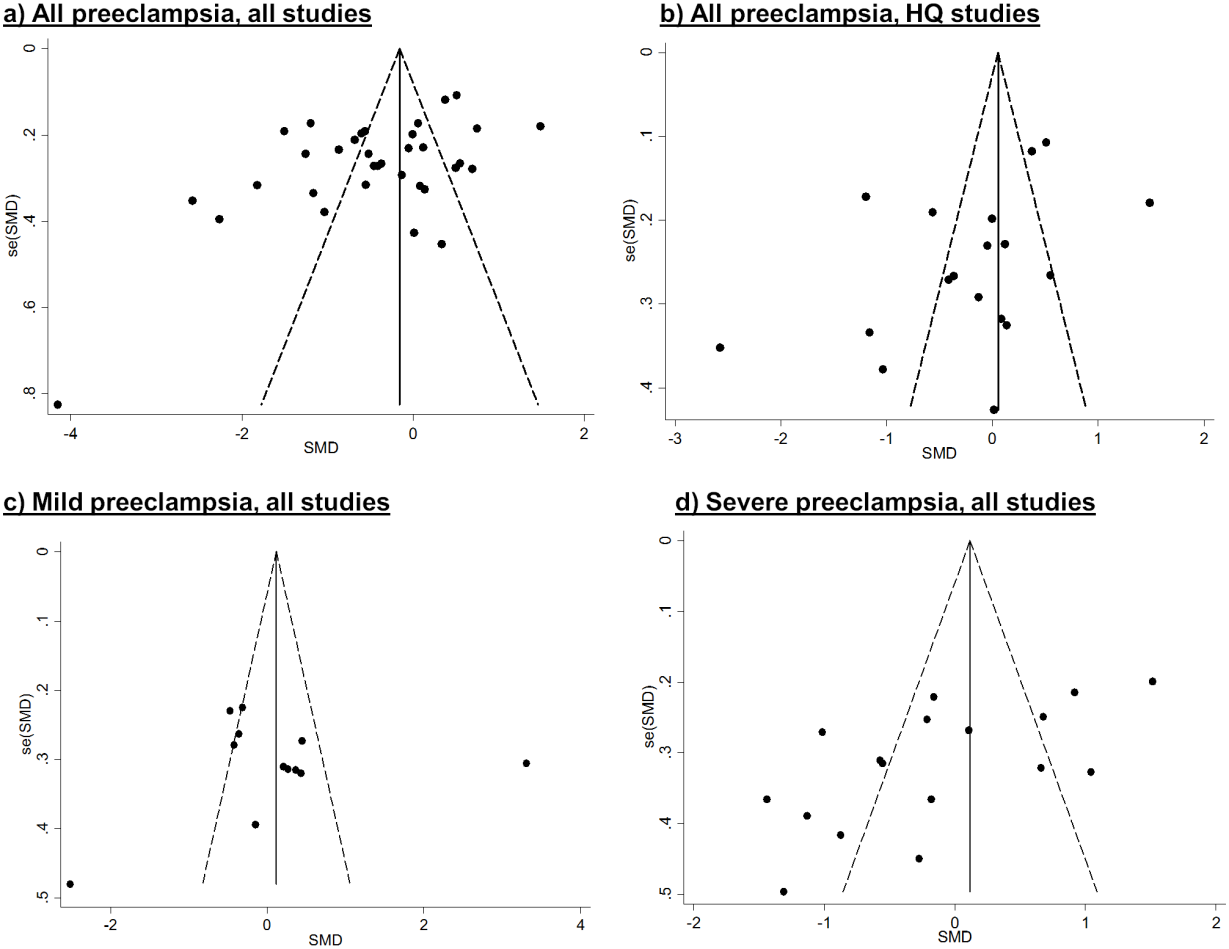
Funnel plots with pseudo-95% confidence intervals for vitamin E studies. High-quality (HQ) studies refer to those that addressed confounding in the design or analysis.

**Fig 5:**
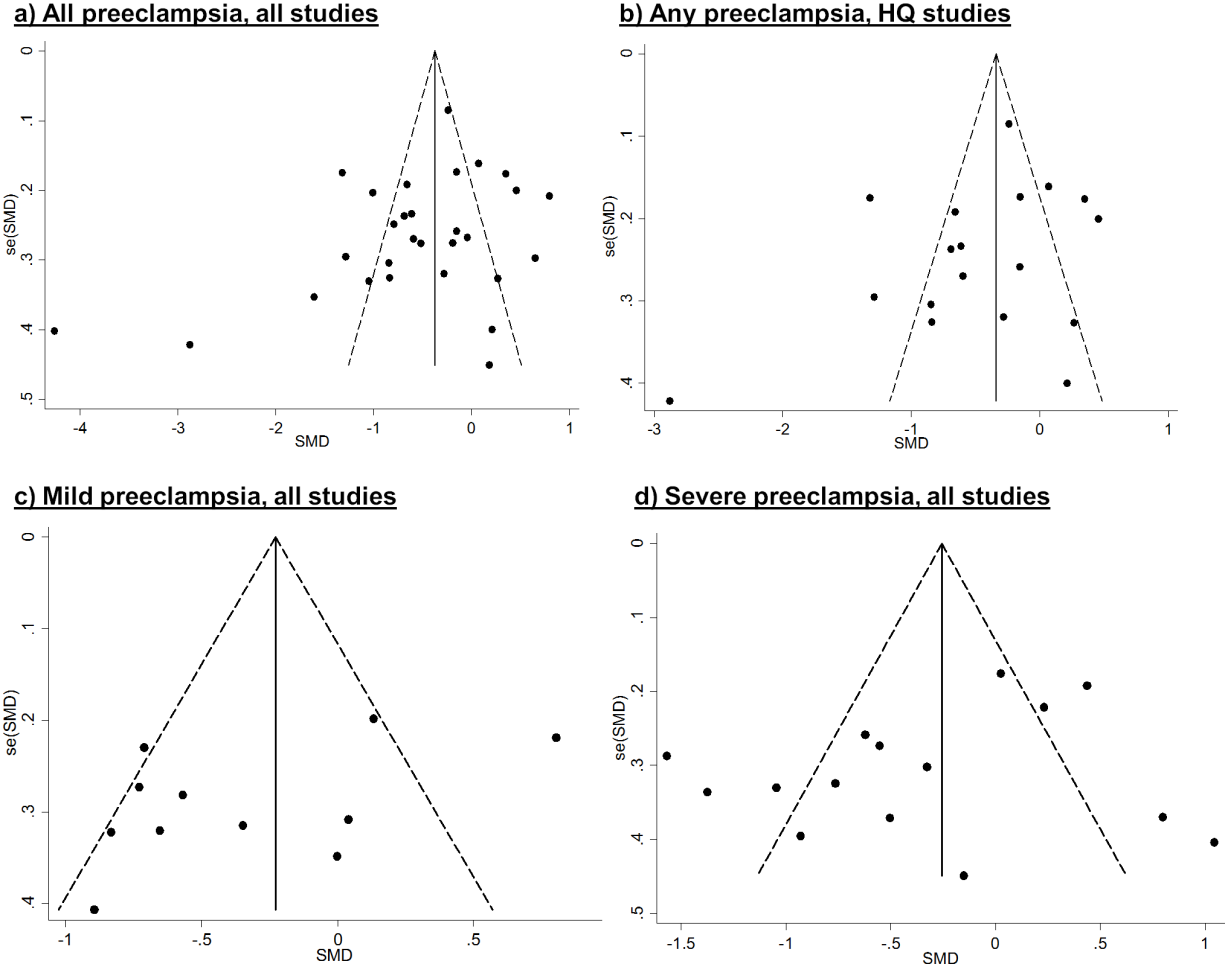
Funnel plots with pseudo-95% confidence intervals for vitamin C studies. High-quality (HQ) studies refer to those that addressed confounding in the design or analysis.

Publication bias was evident among studies of vitamin E and C and all preeclampsia; many of the point estimates fell outside of the pseudo 95% confidence limits (i.e. limits assuming the pooled point estimate is the true association) in both directions. However, among higher-quality studies that addressed confounding (and the subset that measured α-tocopherol, not shown), publication bias appears to be less problematic. Publication bias was less evident among studies of mild preeclampsia, but could not be excluded in studies of severe preeclampsia or eclampsia, since very few of the studies fell on the right side of the funnels (Figs 4 and 5). The funnel plot for retinol suggested publication bias but there were few data points (not shown).

## Discussion

Our systematic review identified a large number of studies that assessed levels of various antioxidants during pregnancy in relation to preeclampsia, and fewer that assessed an association with SGA. With some exceptions, the studies were generally small (less than 50 cases) with high risk of bias. The majority of included studies compared antioxidant levels in women with clinically manifest preeclampsia to third trimester controls. Meta-analyses revealed heterogeneous evidence for reduced third trimester levels of vitamins A, C, E, and carotenoids in preeclampsia. However, confounding was inherent in the crude comparisons that were drawn. Publication bias was also apparent. There was little evidence for differences in antioxidant levels that predated preeclampsia diagnosis. In SGA, there was some limited evidence for reduced levels of vitamin C from the second trimester and lower carotenoid levels in the second trimester.

Several systematic reviews of RCTs have concluded that antioxidant supplementation is not associated with a significant reduction in the risk of preeclampsia or SGA, and may be associated with an increased risk of preterm birth and miscarriage [66–68]. However, the RCTs conducted to date studied a small set of antioxidant interventions (mostly 1000 mg vitamin C and 400 international units vitamin E daily), mainly in women at high risk of preeclampsia. The results of this systematic review may undermine the premise that vitamins C and E are consistently lower in women who develop preeclampsia (and perhaps SGA), on which clinical trials of vitamin C and E for prevention were predicated. Our results suggest that publication bias and confounding may have played a role in forming this premise. We identified significantly negative SMDs for vitamins C and E and preeclampsia, but the findings were very heterogeneous, null for the subset of studies that stratified mild and severe PE, and null for lipid-corrected vitamin E. There was also limited evidence that vitamin C and E levels are reduced in the first or second trimester in pregnancies resulting in preeclampsia or SGA. Among studies that measured other antioxidant levels prospectively, some suggest that differences in vitamins E and A may arise later in pregnancy. Hence, we must consider whether these results, in combination with evidence of a lack of a protective effect of vitamins C and E in RCTs, suggest that lower antioxidant levels may be a consequence, rather than a cause, of these conditions.

While the meta-analysis of any vitamin E showed a negative SMD in preeclampsia cases vs. controls, the meta-analysis of lipid-corrected vitamin E showed no difference. The literature has suggested that correction for cholesterol levels in analyses of tocopherols is appropriate as this may better reflect the potential impact of these antioxidants on reducing oxidative damage [69]. However, women with preeclampsia have been shown to have significantly altered blood lipid measurements [70]. This correction could therefore introduce bias; conversely, failure to adjust may also be problematic. More research on prospectively measured vitamin E levels is needed; preferably with both crude and lipid-corrected levels presented so that is the impact of lipid differences can be better understood.

Our review follows a systematic review published in 2009 that focused on markers of lipid peroxidation and antioxidant levels [28]. Compared to those findings, our pooled SMDs were closer to the null. We believe our approach was more comprehensive and inclusive, since the previous review did not include any prospective studies, and focused more narrowly on vitamins C and E. Further, many new studies have been published since the previous review. We made an effort to explain the heterogeneity observed, even though much of the heterogeneity remained unexplained.

RCTs to date have mainly focused on vitamins C and E. The evidence presented here does not show that vitamins C or E are consistently low in preeclampsia. Other interventions might be beneficial. Our results suggest that carotenes may be an alternative intervention; however, unless future studies show that levels are truly lower before the onset of clinical disease, the evidence is insufficient to recommend carotene supplementation to prevent preeclampsia. We also found evidence that total carotenoids are lower in the second trimester in SGA. Few studies measured one or more carotenoids in preeclampsia, and more data are needed to assess whether supplementation with carotenoid antioxidants could be an effective intervention to prevent preeclampsia or SGA.

Few studies included in our review addressed confounding at the design or analysis stage. Most studies reported only means and standard deviations and a t-test for differences. Thus, confounding likely explains at least part of the crude associations observed. Some studies used regression models to adjust for confounding. Studies that reported both crude and adjusted ORs allow a more in-depth consideration of the extent of confounding. When the data were not presented in a way amenable to statistical pooling of adjusted effect estimates, we tried to summarize the results qualitatively. We also assessed whether excluding studies that did not address confounding affected the point estimate and our assessment of publication bias. We found that the SMD was shifted toward the null, with less evidence of publication bias.

Inclusion and exclusion criteria of individual studies differed substantially. Some studies included only women at high risk for preeclampsia, while others excluded women who had risk factors such as pre-existing hypertension, diabetes, or multiple pregnancies. We investigated heterogeneity in the study results due to differences in study populations, but we were limited in number of comparisons we could make. Nevertheless, the population studied was not significantly associated with the pooled SMD.

Most of the preeclampsia studies measured antioxidants in late pregnancy, in most cases, after the clinical diagnosis. Hence, we are unable to infer whether differences observed predated clinical signs and participated in a causal mechanism. Only one study stratified the cases by the timing of preeclampsia, early (<34 weeks) vs. late, which is a widely accepted subgrouping that may represent distinct conditions with different causal mechanisms [71]. We could not exclude the possibility that antioxidant levels were affected by preeclampsia itself or treatments used in these women.

Due to limited resources available, we could not translate studies in languages other than English and French. However, we searched many databases without language restrictions. Only 5% (7 of 135) of the studies we judged as potentially eligible based on a translated title and abstract were published in other languages. However, we acknowledge that at least a few relevant studies could have been missed by the language restrictions we applied.

Very few studies provided adjusted ORs for the association in which we are interested: the risk/odds of preeclampsia or SGA according to level of antioxidant. Given the data available to us, we calculated the pooled SMD, which provides a measure of the average difference (in terms of SD units) between study groups. Our interpretation is hence limited, because each of the pooled SMD estimates are likely confounded to some degree. Studies that accounted for confounding in the design by matching or by restriction are less likely to be confounded.

We did not consider studies of other micronutrients that act as co-factors to antioxidant enzymes (e.g. selenium, zinc) and that may also modulate oxidative stress in preeclampsia and SGA. Several studies have looked at whether levels of these micronutrients are differential in preeclamptic pregnancies [72, 73], and clinical trials of selenium supplementation have been reported [74]. We did not consider all biomolecules with antioxidant properties which could potentially be given as dietary supplements (e.g. melatonin). While these were outside of the scope of this review, they may be relevant to explore in future systematic reviews.

We did not exclude studies on the basis of research design or population. Hence, we could investigate sources of heterogeneity in the literature and seek to understand if differences in these factors led to different conclusions. We carried out an exhaustive search strategy developed in consultation with an experienced librarian that covered a wide time range and included many databases. We also carefully reviewed the references of included studies and review articles to identify any studies we may have missed. The entire review process was carried out in duplicate. The reviewers also met early in each stage to review whether they were interpreting eligibility criteria in the same way and whether the data extraction process was consistent.

Our review assessed many relevant markers and included prospective studies so that differences in early- to mid-pregnancy could be investigated, in addition to evaluating whether consistent differences were apparent in late pregnancy. We were able to determine that there was limited high-quality evidence for systematic differences in antioxidant levels before clinical detection of preeclampsia or recognition of SGA birth. We believe that such evidence would be needed to justify future trials of any antioxidant supplementation intervention.

## Conclusion

A large number of studies have measured non-enzymatic antioxidant levels in maternal blood during pregnancy in relation to preeclampsia and several have assessed them in relation to SGA. The results for crude differences between cases and controls, as assessed by the pooled SMD showed a tendency toward lower vitamin E and C levels in cases of preeclampsia and SGA, and lower vitamin A in preeclampsia, but substantial heterogeneity of results suggests the need for cautious interpretation. Some of this heterogeneity was explained by assay methods, including HPLC and fasting status. Many of the studies were of low quality, and confounding and publication bias are potentially problematic. Future observational studies with a sufficiently large sample size and high methodologic quality are needed to determine if antioxidant levels are low at a causally-relevant time period, before clinical manifestation of preeclampsia or SGA, when intervening on these levels may change the pregnancy outcome. Studies with serial measures are also of interest to more clearly identify when differences may arise. The results of our review do not suggest an imminent need for new clinical trials since it is unclear what interventions may work best and in what populations. Substantial unexplained heterogeneity in findings among the studies we reviewed limits our ability to know which populations might benefit from future interventions. Future observational studies may clarify these issues and lead the way for clinical trials of promising interventions that have not yet been tested.

## Supporting Information

S1 Checklist (DOC)

S1 File Appendix A: Complete PubMed Search; Appendix B: Manual for risk of bias assessment; Appendix C: Details of methods used to combine study groups and description of pooling; Appendix D: Complete list of included studies; Appendix E: Description of data available that was not amenable to statistical pooling; Appendix F: Additional details of study results not meta-analyzed; Appendix G: Odds ratios reported in the reviewed studies for vitamin E and preeclampsia; Appendix H: Results of influence analyses (for meta-analyses with 10 or more observations); Appendix I: Tables from additional meta-regression analyses.
(PDF)

S1 Protocol Systematic review and meta-analysis protocol prospectively registered in the PROSPERO database;www.crd.york.ac.uk/PROSPERO (registration no. CRD42013003519). (PDF)

S1 Text Meta-analysis output showing individual study estimates of association and pooled results. (TXT)
