# Supplementary material for: Maternal Antioxidant Levels in Pregnancy and Risk of Preeclampsia and Small for Gestational Age Birth: A Systematic Review and Meta-Analysis
Source: PLoS One. 2015 Aug 6;10(8):e0135192. doi: 10.1371/journal.pone.0135192 (PMC4527773; doi:10.1371/journal.pone.0135192)
Supplement: S1 File — Appendix A: Complete PubMed Search; Appendix B: Manual for risk of bias assessment; Appendix C: Details of methods used to combine study groups and description of pooling; Appendix D: Complete list of included studies; Appendix E: Description of data available that was not amenable to statistical pooling; Appendix F: Additional details of study results not meta-analyzed; Appendix G: Odds ratios reported in the reviewed studies for vitamin E and preeclampsia; Appendix H: Results of influence analyses (for meta-analyses with 10 or more observations); Appendix I: Tables from additional meta-regression analyses. (PDF) [file pone.0135192.s002.pdf]

S1 Supporting Information

Appendix A: Complete PubMed Search

Appendix B: Manual for risk of bias assessment

Appendix C: Details of methods used to combine study groups and description of pooling

Appendix D: Complete list of included studies

Appendix E: Description of data available that was not amenable to statistical pooling

Appendix F: Additional details of study results not meta-analyzed

Appendix G: Odds ratios reported in the reviewed studies for vitamin E and preeclampsia

Appendix H: Results of influence analyses (for meta-analyses with 10 or more observations)

Appendix I: Tables from additional meta-regression analyses

Appendix A: Complete PubMed Search

Pubmed search conducted January 16, 2013 which was adapted to other databases: (("Antioxidants"[Mesh] OR "Carotenoids"[Mesh] OR "Vitamin A"[Mesh] OR "Ascorbic Acid"[Mesh] OR antioxidant*[tiab] OR anti-oxidant* OR tocopherol*[tiab] OR "vitamin e"[tiab] OR carotenoid*[tiab] OR betacarotene*[tiab] OR beta-carotene*[tiab] OR carotene*[tiab] OR lycopene*[tiab] OR cryptoxanthin*[tiab] OR lutein*[tiab] OR "vitamin a"[tiab] OR retinol[tiab] OR "vitamin c"[tiab] OR ascorbate*[tiab] OR ascorbic acid[tiab]) AND ("Pre-Eclampsia"[Mesh] OR pre-eclampsia[tiab] OR preeclampsia[tiab] OR (toxemia[tiab] AND pregnancy[tiab]) OR "Infant, Small for Gestational Age"[Mesh] OR "small for gestational age"[tiab] OR "small-for-gestational-age"[tiab] OR SGA[tiab] OR "Fetal Growth Retardation"[Mesh] OR "fetal growth retardation"[tiab] OR "fetal growth restriction"[tiab] OR FGR[tiab] OR "intrauterine growth retardation"[tiab] OR "intra-uterine growth retardation"[tiab] OR "intrauterine growth restriction"[tiab] OR "intra-uterine growth restriction"[tiab] OR IUGR[tiab])) NOT animals[Mesh:noexp] AND ("1970/01/01"[PDAT] : "2013/12/31"[PDAT])

Appendix B: Manual for risk of bias assessment

RB1. Was the study clinical setting well-described?
Yes = described clinical setting (teaching vs. non / tertiary care, location)
No = did not describe any
Unclear = missing one key descriptor

RB2. Were incomplete data (i.e. missing data) adequately described?
Yes = described proportion of missing data or described there was no missing data
No = no description of missing data or incomplete information on missing data
Unclear = no mention of missing data

RB3. Were statistical analyses described adequately?
Yes = described all analyses presented in methods section
No = missing important information on analyses
Unclear = some unclear portions of results

RB4. Were analyses appropriate?
Yes = appropriate
No = did not account for matching [if they did individual matching, they should use statistical methods that treat subjects as pairs, e.g. paired t-test, or conditional logistic regression], described distributions as skewed but used a t-test (which assumes normality), etc.
Unclear = not well described or missing key information

RB5. Did analysis provide sufficient presentation of data?
Yes = means and SD or median and IQR in figures or tables or text; AND presented patient characteristics by group (same groups as presented in analysis)
No = did not present characteristics of patients by groups stratified in analyses
Unclear = not well described or missing key covariates

RB6. Is the study report free of the suggestion of selective reporting?
Yes = all exposure-outcome relationships described
No = indication of selective outcome reporting, only select time points / markers
Unclear = judgement (data not shown)

RB7. Was confounding accounted for in the design and/or analysis?
Yes = used restriction or matching [design], stratification or adjustment [analysis] to control for confounding
No = did not account for confounding (only presented crude results)
Unclear = concern of residual confounding or unclear description of methods used

Appendix C: Details of methods used to combine study groups and description of pooling

Table 3.1: Description of case groups included and pooled by meta-analysis

**Meta-analysis**	**Case groups pooled**
All preeclampsia<br>Includes any, mild, severe, superimposed, early-onset, late-onset preeclampsia, eclampsia	Mild & severe<br>Preeclampsia & eclampsia<br>Preeclampsia & superimposed preeclampsia<br>Mild & severe & eclampsia
Mild preeclampsia	None
Severe preeclampsia<br>Includes eclampsia	Severe & eclampsia

Table 3.2: Equations used to pool means and standard deviations across study case groups*

	**Group 1 (e.g. mild)**	**Group 2 (e.g. severe)**	**Combined groups**
**Sample size**	N_1	N_2	$N_1 + N_2$
**Mean**	M_1	M_2	$\frac{N_1 M_1 + N_2 M_2}{N_1 + N_2}$
**SD**	SD_1	SD_2	$\sqrt{\frac{(N_1 - 1)SD_1^2 + (N_2 - 1)SD_2^2 + \frac{N_1 N_2}{N_1 + N_2}(M_1^2 + M_2^2 - 2M_1 M_2)}{N_1 + N_2 - 1}}$

*Adapted with permission from Table7.7.a in Higgins JPT, Deeks JJ (editors). Chapter 7: Selecting studies and collecting data. In: Higgins JPT, Green S (editors), Cochrane Handbook for Systematic Reviews of Interventions Version 5.1.0 (updated March 2011). The Cochrane Collaboration, 2011. Available from http://handbook.cochrane.org/.

<u>Stata code for program for combining groups</u>

```
program combgroups, rclass
        version 11
        args n1 n2 m1 m2 sd1 sd2
        scalar n_comb=`n1'+`n2'
        scalar mean_comb=( [(`n1'*`m1') + (`n2'*`m2')]  / (`n1'+`n2') )
        scalar sd_comb= sqrt( ( [(`n1'-1)*`sd1'^2 ] + [(`n2'-1)*`sd2'^2 ] +
        [(`n1'*`n2')/(`n1'+`n2')] * [`m1'^2+`m2'^2-(2*`m1'*`m2')] ) / [`n1'+`n2'-1] )

        display "Combined N = " `n1'+`n2'
        display "Combined Mean = " ( [(`n1'*`m1') + (`n2'*`m2')]  / (`n1'+`n2') )
        display "Combined SD = " sqrt( ( [(`n1'-1)*`sd1'^2 ] + [(`n2'-1)*`sd2'^2 ] +
        [(`n1'*`n2')/(`n1'+`n2')] * [`m1'^2+`m2'^2-(2*`m1'*`m2')] ) / [`n1'+`n2'-1] )

        return scalar n=n_comb
        return scalar mean=mean_comb
        return scalar sd=sd_comb

        end
```

Appendix D: Complete list of included studies

Agarwal K, Dabke AT, Phuljhele NL, and Khandwal OP. Factors affecting serum vitamin A levels in matched maternal-cord pairs. *Indian journal of pediatrics* 2008: **75**; 443-446.

Akyol D, Mungan T, Gorkemli H, and Nuhoglu G. Maternal levels of vitamin E in normal and preeclamptic pregnancy. *Arch Gynecol Obstet* 2000: **263**; 151-155.

Azar M, Basu A, Jenkins AJ, Nankervis AJ, Hanssen KF, Scholz H, Henriksen T, Garg SK, Hammad SM, Scardo JA, *et al.* Serum carotenoids and fat-soluble vitamins in women with type 1 diabetes and preeclampsia: a longitudinal study. *Diabetes care* 2011: **34**; 1258-1264.

Bakheit KH, Ghebremeskel K, Zaiger G, Elbashir MI, and Adam I. Erythrocyte antioxidant enzymes and plasma antioxidant vitamins in Sudanese women with pre-eclampsia. *Journal of obstetrics and gynaecology : the journal of the Institute of Obstetrics and Gynaecology* 2010: **30**; 147-150.

Basu RJS and Arulanantham R. A study of serum protein and retinol levels in pregnancy and toxaemia of pregnancy in women of low socio economic status. *Indian Journal of Medical Research* 1973: **61 (4)**; 589-595.

Ben-Haroush A, Harell D, Hod M, Bardin R, Kaplan B, Orvieto R, and Bar J. Plasma levels of vitamin E in pregnant women prior to the development of preeclampsia and other hypertensive complications. *Gynecologic and obstetric investigation* 2002: **54**; 26-30.

Bowen RS, Mars M, Chuturgoon AA, Dutton MF, and Moodley J. The response of the dietary anti-oxidants vitamin E and vitamin C to oxidative stress in pre-eclampsia. *Journal of obstetrics and gynaecology : the journal of the Institute of Obstetrics and Gynaecology* 1998: **18**; 9-13.

Bowen RS, Moodley J, Dutton MF, and Theron AJ. Oxidative stress in pre-eclampsia. *Acta Obstet Gynecol Scand* 2001: **80**; 719-725.

Chappell LC, Seed PT, Briley A, Kelly FJ, Hunt BJ, Charnock-Jones DS, Mallet AI, and Poston L. A longitudinal study of biochemical variables in women at risk of preeclampsia. *American Journal of Obstetrics and Gynecology* 2002: **187**; 127-136.

Dehghan MH, Daryani A, and Dehghanan R. Homeostasis status between prooxidants and antioxidants as a potent marker in Iranian preeclamptic patients. *Saudi medical journal* 2007: **28**; 696-700.

Dirican M, Safak O, Uncu G, and Sarandol E. Susceptibility of red blood cell lipids to in vitro oxidation and antioxidant status in preeclampsia. *European journal of obstetrics, gynecology, and reproductive biology* 2008: **140**; 158-164.

Dreyfuss ML, Msamanga GI, Spiegelman D, Hunter DJ, Urassa EJ, Hertzmark E, and Fawzi WW. Determinants of low birth weight among HIV-infected pregnant women in Tanzania. *The American journal of clinical nutrition* 2001: **74**; 814-826.

Elsen C, Rivas-Echeverria C, Sahland K, Sanchez R, Molma L, Pahl L, Wallinger R, Volz J, Wacker J, and Fruhauf J. Vitamins E, A and B as possible risk factors for preeclampsia under

consideration of the PROPER study ("prevention of preeclampsia by high-dose riboflavin supplementationo"). *Geburtshilfe und Frauenheilkunde* 2012: **72 (9)**; 846-852.

Gratacos E, Casals E, Deulofeu R, Gomez O, Cararach V, Alonso PL, and Fortuny A. Serum and placental lipid peroxides in chronic hypertension during pregnancy with and without superimposed preeclampsia. *Hypertension in pregnancy : official journal of the International Society for the Study of Hypertension in Pregnancy* 1999: **18**; 139-146.

Harma M and Erel O. Measurement of the total antioxidant response in preeclampsia with a novel automated method. *European journal of obstetrics, gynecology, and reproductive biology* 2005: **118**; 47-51.

Harsem NK, Braekke K, and Staff AC. Augmented oxidative stress as well as antioxidant capacity in maternal circulation in preeclampsia. *European journal of obstetrics, gynecology, and reproductive biology* 2006: **128**; 209-215.

Harsem NK, Braekke K, Torjussen T, Hanssen K, and Staff AC. Advanced glycation end products in pregnancies complicated with diabetes mellitus or preeclampsia. *Hypertension in pregnancy : official journal of the International Society for the Study of Hypertension in Pregnancy* 2008: **27**; 374-386.

Howlader ZH, Kabir Y, Khan TA, Islam R, Begum F, and Huffman FG. Plasma lipid profile, lipid peroxidation and antioxidant status in preeclamptic and uncomplicated pregnancies in Bangladesh. *Journal of Medical Sciences* 2007: **7 (8)**; 1276-1282.

Hubel CA, Kagan VE, Kisin ER, McLaughlin MK, and Roberts JM. Increased ascorbate radical formation and ascorbate depletion in plasma from women with preeclampsia: implications for oxidative stress. *Free radical biology & medicine* 1997: **23**; 597-609.

Ikpen MA, Eigbefoh J, Eifediyi RA, Isabu PA, Okogbenin S, Okogbo FO, Momoh M, and Ekwedigwe KC. Determination of antioxidant status of pre-eclamptic and normotensive sub-rural Nigerian pregnant women at the Irrua Specialist Teaching Hospital, Irrua, Edo State. *The journal of maternal-fetal & neonatal medicine : the official journal of the European Association of Perinatal Medicine, the Federation of Asia and Oceania Perinatal Societies, the International Society of Perinatal Obstetricians* 2012: **25**; 2046-2050.

Islam SN, Jr., Ahsan T, Khatun S, Khan MN, and Ahsan M. Serum vitamin E, C and A status in pre-eclampsia and eclampsia patients, and their correlation with blood pressure: a study in Dhaka, Bangladesh. *Malaysian journal of nutrition* 2004: **10**; 207-214.

Jendryczko A and Drozdz M. Plasma retinol, beta-carotene and vitamin E levels in relation to the future risk of pre-eclampsia. *Zentralblatt fur Gynakologie* 1989: **111**; 1121-1123.

Kaur G, Mishra S, Sehgal A, and Prasad R. Alterations in lipid peroxidation and antioxidant status in pregnancy with preeclampsia. *Mol Cell Biochem* 2008: **313**; 37-44.

Kerver JM, Holzman CB, Tian Y, Shroff MR, and Evans RW. Maternal Serum Antioxidant Vitamins and Pregnancy Outcomes. *FASEB Journal* 2012: **26**.

Kharb S. Vitamin E and C in preeclampsia. *European journal of obstetrics, gynecology, and reproductive biology* 2000: **93**; 37-39.

Kiondo P, Tumwesigye NM, Wandabwa J, Wamuyu-Maina G, Bimenya GS, and Okong P. Plasma vitamin C assay in women of reproductive age in Kampala, Uganda, using a colorimetric method. *Tropical medicine & international health : TM & IH* 2012: **17**; 191-196.

Kolusari A, Kurdoglu M, Yildizhan R, Adali E, Edirne T, Cebi A, Demir H, and Yoruk IH. Catalase activity, serum trace element and heavy metal concentrations, and vitamin A, D and E levels in pre-eclampsia. *The Journal of international medical research* 2008: **36**; 1335-1341.

Llurba E, Gratacos E, Martin-Gallan P, Cabero L, and Dominguez C. A comprehensive study of oxidative stress and antioxidant status in preeclampsia and normal pregnancy. *Free radical biology & medicine* 2004: **37**; 557-570.

Madazli R, Benian A, Gumustas K, Uzun H, Ocak V, and Aksu F. Lipid peroxidation and antioxidants in preeclampsia. *European journal of obstetrics, gynecology, and reproductive biology* 1999: **85**; 205-208.

Mehendale S, Kilari A, Dangat K, Taralekar V, Mahadik S, and Joshi S. Fatty acids, antioxidants, and oxidative stress in pre-eclampsia. *Int J Gynaecol Obstet* 2008: **100**; 234-238.

Mikhail MS, Anyaegbunam A, Garfinkel D, Palan PR, Basu J, and Romney SL. Preeclampsia and antioxidant nutrients: decreased plasma levels of reduced ascorbic acid, alpha-tocopherol, and beta-carotene in women with preeclampsia. *American Journal of Obstetrics and Gynecology* 1994: **171**; 150-157.

Mohanty S, Sahu PK, Mandal MK, Mohapatra PC, and Panda A. Evaluation of oxidative stress in pregnancy induced hypertension. *Indian journal of clinical biochemistry : IJCB* 2006: **21**; 101-105.

Mohindra A, Kabi BC, Kaul N, and Trivedi SS. Vitamin E and carotene status in pre-eclamptic pregnant women from India. *Panminerva medica* 2002: **44**; 261-264.

Morris JM, Gopaul NK, Endresen MJ, Knight M, Linton EA, Dhir S, Anggard EE, and Redman CW. Circulating markers of oxidative stress are raised in normal pregnancy and pre-eclampsia. *British journal of obstetrics and gynaecology* 1998: **105**; 1195-1199.

Mutlu-Turkoglu U, Ademoglu E, Ibrahimoglu L, Aykac-Toker G, and Uysal M. Imbalance between lipid peroxidation and antioxidant status in preeclampsia. *Gynecologic and obstetric investigation* 1998: **46**; 37-40.

Nilar W, San Aye K, Myo W, and Aye Aye M. Plasma malondialdehyde, vitamin C and vitamin E levels of normal pregnant and preeclamptic pregnant women in West Yangon General Hospital. *Myanmar Health Sciences Research Journal* 2009: **21**; 152-156.

Noyan T, Guler A, Sekeroglu MR, and Kamaci M. Serum advanced oxidation protein products, myeloperoxidase and ascorbic acid in pre-eclampsia and eclampsia. *The Australian & New Zealand journal of obstetrics & gynaecology* 2006: **46**; 486-491.

Ortega-Senovilla H, Alvino G, Taricco E, Cetin I, and Herrera E. Enhanced circulating retinol and non-esterified fatty acids in pregnancies complicated with intrauterine growth restriction. *Clinical science* 2010: **118**; 351-358.

Ozan H, Esmer A, Kolsal N, Copur OU, and Ediz B. Plasma ascorbic acid level and erythrocyte fragility in preeclampsia and eclampsia. *European journal of obstetrics, gynecology, and reproductive biology* 1997: **71**; 35-40.

Palan PR, Mikhail MS, and Romney SL. Placental and serum levels of carotenoids in preeclampsia. *Obstetrics and gynecology* 2001: **98**; 459-462.

Palan PR, Shaban DW, Martino T, and Mikhail MS. Lipid-soluble antioxidants and pregnancy: maternal serum levels of coenzyme Q10, alpha-tocopherol and gamma-tocopherol in preeclampsia and normal pregnancy. *Gynecologic and obstetric investigation* 2004: **58**; 8-13.

Panburana P, Phuapradit W, and Puchaiwatananon O. Antioxidant nutrients and lipid peroxide levels in Thai preeclamptic pregnant women. *J Obstet Gynaecol Res* 2000: **26**; 377-381.

Rajasingam D, Seed PT, Briley AL, Shennan AH, and Poston L. A prospective study of pregnancy outcome and biomarkers of oxidative stress in nulliparous obese women. *American Journal of Obstetrics and Gynecology* 2009: **200**; 395 e391-399.

Roland L, Gagne A, Belanger MC, Boutet M, Berthiaume L, Fraser WD, Julien P, and Bilodeau JF. Existence of compensatory defense mechanisms against oxidative stress and hypertension in preeclampsia. *Hypertension in pregnancy : official journal of the International Society for the Study of Hypertension in Pregnancy* 2010: **29**; 21-37.

Sagol S, Ozkinay E, and Ozsener S. Impaired antioxidant activity in women with pre-eclampsia. *Int J Gynaecol Obstet* 1999: **64**; 121-127.

Saker M, Soulimane Mokhtari N, Merzouk SA, Merzouk H, Belarbi B, and Narce M. Oxidant and antioxidant status in mothers and their newborns according to birthweight. *European journal of obstetrics, gynecology, and reproductive biology* 2008: **141**; 95-99.

Schiff E, Friedman SA, Stampfer M, Kao L, Barrett PH, and Sibai BM. Dietary consumption and plasma concentrations of vitamin E in pregnancies complicated by preeclampsia. *American Journal of Obstetrics and Gynecology* 1996: **175**; 1024-1028.

Scholl TO, Chen X, Sims M, and Stein TP. Vitamin E: maternal concentrations are associated with fetal growth. *The American journal of clinical nutrition* 2006: **84**; 1442-1448.

Serdar Z, Gur E, Colakoethullary M, Develioethlu O, and Sarandol E. Lipid and protein oxidation and antioxidant function in women with mild and severe preeclampsia. *Arch Gynecol Obstet* 2003: **268**; 19-25.

Serdar Z, Gur E, Develioglu O, Colakogullari M, and Dirican M. Placental and decidual lipid peroxidation and antioxidant defenses in preeclampsia - Lipid peroxidation in preeclampsia. *Pathophysiology* 2002: **9 (1)**; 21-25.

Sharma JB, Sharma A, Bahadur A, Vimala N, Satyam A, and Mittal S. Oxidative stress markers and antioxidant levels in normal pregnancy and pre-eclampsia. *Int J Gynaecol Obstet* 2006: **94**; 23-27.

Sharma SC, Sabra A, Molloy A, and Bonnar J. Comparison of blood levels of histamine and total ascorbic acid in pre-eclampsia with normal pregnancy. *Human nutrition. Clinical nutrition* 1984: **38**; 3-9.

Suhail M and Faizul-Suhail M. Lipoperoxidation and its correlation with antioxidant vitamins in non-pregnant, pregnant, and preeclamptic women. *Journal of Chinese Clinical Medicine* 2009: **4 (1)**; 19-25.

Uotila JT, Kirkkola AL, Rorarius M, Tuimala RJ, and Metsa-Ketela T. The total peroxyl radical-trapping ability of plasma and cerebrospinal fluid in normal and preeclamptic parturients. *Free radical biology & medicine* 1994: **16**; 581-590.

Uotila JT, Tuimala RJ, Aarnio TM, Pyykko KA, and Ahotupa MO. Findings on lipid peroxidation and antioxidant function in hypertensive complications of pregnancy. *British journal of obstetrics and gynaecology* 1993: **100**; 270-276.

Wei SQ, Julien P, Luo ZC, Audibert F, and Fraser W. Maternal plasma beta-carotene, ICAM and VCAM levels in normal and preeclamptic pregnancies. *American journal of epidemiology* 2012: **175 (Suppl 11)**; S79.

Wikstrom AK, Nash P, Eriksson UJ, and Olovsson MH. Evidence of increased oxidative stress and a change in the plasminogen activator inhibitor (PAI)-1 to PAI-2 ratio in early-onset but not late-onset preeclampsia. *American Journal of Obstetrics and Gynecology* 2009: **201**; 597 e591-598.

Williams MA, Woelk GB, King IB, Jenkins L, and Mahomed K. Plasma carotenoids, retinol, tocopherols, and lipoproteins in preeclamptic and normotensive pregnant Zimbabwean women. *American journal of hypertension* 2003: **16**; 665-672.

Xu H. Maternal Nutrition and The Risk of Preeclampsia. 2011. Universite de Montreal (Canada), Canada, pp 331.

Yanik FF, Amanvermez R, Yanik A, Celik C, and Kokcu A. Pre-eclampsia and eclampsia associated with increased lipid peroxidation and decreased serum vitamin E levels. *International Journal of Gynecology and Obstetrics* 1999: **64 (1)**; 27-33.

Zhang C, Luthy D, King I, Walsh S, Sorensen T, Kestin M, and Williams M. Maternal first-trimester plasma ascorbic acid (vitamin C) concentrations in relation to risk of preeclampsia. *American Journal of Obstetrics & Gynecology* 2001: **184**; S77.

Zhang C, Williams MA, Sanchez SE, King IB, Ware-Jauregui S, Larrabure G, Bazul V, and Leisenring WM. Plasma concentrations of carotenoids, retinol, and tocopherols in preeclamptic and normotensive pregnant women. *American journal of epidemiology* 2001: **153**; 572-580.

Ziari SA, Mireles VL, Cantu CG, Cervantes M, 3rd, Idrisa A, Bobsom D, Tsin AT, and Glew RH. Serum vitamin A, vitamin E, and beta-carotene levels in preeclamptic women in northern nigeria. *American journal of perinatology* 1996: **13**; 287-291.

Zusterzeel PL, Steegers-Theunissen RP, Harren FJ, Stekkinger E, Kateman H, Timmerman BH, Berkelmans R, Nieuwenhuizen A, Peters WH, Raijmakers MT, *et al.* Ethene and other biomarkers of oxidative stress in hypertensive disorders of pregnancy. *Hypertension in pregnancy : official journal of the International Society for the Study of Hypertension in Pregnancy* 2002: **21**; 39-49.

Appendix E: Description of data available that was not amenable to statistical pooling

Study	Antioxidant	Data provided that prevented statistical pooling
Azar et al. (2011)	Lipid-corrected vitamin E<br>α-carotene<br>β-carotene<br>lycopene	Geometric means (in figures)*
Bakheit et al. (2010)	Vitamin E<br>Retinol	Median and interquartile range
Chappell et al. (2002)	Lipid-corrected vitamin E	Geometric means (in figures)
Dirican et al. (2008)	Lipid-corrected vitamin E	None, result described in text
Harsem, Braekke, and Staff (2006)	Vitamin E	Median and 95% confidence interval
Harsem et al. (2008)	Vitamin E	Median and 95% confidence interval
Hubel et al. (1997)	Vitamin E<br>Lipid-corrected vitamin E<br>Vitamin C	Median and interquartile range
Palan et al. (2004)	Vitamin E	Median and 10th to 90th percentiles
Rajasingam et al. (2009)	Lipid-corrected vitamin E	None, result described in text
Serdar et al. (2003)	Vitamin E<br>Total carotene	ORs across tertiles ; only mean (SD) for lipid-corrected form
Wei et al. (2012)	β-carotene	OR for lowest versus highest quartile
Zusterzeel et al. (2002)	Vitamin E<br>Vitamin C	Median and interquartile range

*The authors provided arithmetic means for retinol, α-tocopherol, and lutein; hence we could meta-analyzed the data extracted from figures for these markers. We were unable to pool the geometric means for γ-tocopherol, α-carotene, β-carotene, lycopene, α-tocopherol/total lipids, γ-tocopherol/total lipids

Appendix F: Additional details of study results not meta-analyzed

Vitamin E

Vitamin E (α-tocopherol, total tocopherol, or unspecified) and preeclampsia

First/Second Trimester (5 studies)

One study measured vitamin E in the first trimester among women with diabetes and found no significant difference for vitamin E or lipid-corrected vitamin E between subjects who subsequently developed preeclampsia and controls (Azar et al. 2011). Five studies measured vitamin E in the second trimester (Azar et al. 2011; Ben-Haroush et al. 2002; Chappell et al. 2002; Rajasingam et al. 2009; Xu 2011). Four of the five studies reported no significant difference between cases and controls in vitamin E (Ben-Haroush et al. 2002; Xu 2011), or lipid-corrected vitamin E (Chappell et al. 2002; Rajasingam et al. 2009). Only the study by Azar et al. (2011) reported finding a significantly higher level of vitamin E among preeclampsia cases compared to controls; however, no difference was observed for lipid-corrected vitamin E.

Third Trimester (41 studies)

Among four other studies not included in this meta-analysis for reasons provided in Appendix 5, two reported similar levels in cases and controls (Harsem, Braekke, and Staff 2006; Harsem et al. 2008), and two reported higher levels of vitamin E in cases (Bakheit et al. 2010; Hubel et al. 1997); and in one, the difference was statistically significant.(Bakheit et al. 2010) Two studies we were unable to pool reported significantly lower vitamin E in the third trimester for both mild and severe preeclampsia (Palan et al. 2004; Serdar et al. 2003), and one study found nonsignificantly higher levels in mild preeclampsia (Zusterzeel et al. 2002). Among four studies of preeclampsia that we were unable to pool, none reported an association between lipid-adjusted vitamin E measured in the third trimester and any preeclampsia (Hubel et al. 1997; Chappell et al. 2002; Azar et al. 2011) or mild/severe preeclampsia (Dirican et al. 2008).

γ-Tocopherol and preeclampsia

First/Second Trimester (3 studies)

Azar et al. (2011) found γ-tocopherol to be non-significantly higher in all three trimesters among women who developed preeclampsia, and correction for total lipids did not alter the findings. Rajasingam et al. (2009) reported that γ-tocopherol corrected for total cholesterol was similar at 14-22 weeks between women who would and would not later develop preeclampsia in a cohort of 385 high-risk (nulliparous, obese) women. The larger study by Xu (2011) found significantly elevated uncorrected γ-tocopherol at 12-18 weeks; the adjusted OR per 1 unit SD was 1.35 (1.02, 1.78).

Third Trimester (5 studies)

Among five studies that measured γ-tocopherol in the third trimester, none identified significant differences between cases of mild or severe preeclampsia and controls (adjusted for apoB) (Palan et al. 2004), or of any preeclampsia and controls (Zhang, Williams, et al. 2001; Williams et al. 2003; Roland et al. 2010; Azar et al. 2011). One of two large case-control studies observed a trend toward higher odds of preeclampsia among those in higher quartiles of uncorrected γ-tocopherol, even after adjustment for confounding (Williams et al. 2003).

Vitamin E (α-tocopherol, total tocopherol, or unspecified) and SGA (8 studies)

Two studies reported that vitamin E was significantly lower in the second trimester for pregnancies that resulted in an SGA birth vs. controls (Dreyfuss et al. 2001; Kerver et al. 2012); however, one reported that after adjusting for total cholesterol, the levels of lipid-adjusted vitamin E were similar between the groups (Kerver et al. 2012). This is consistent with the findings of three other studies which found no significant difference in the second trimester for lipid-adjusted vitamin E (total cholesterol) (Rajasingam et al. 2009; Scholl et al. 2006; Chappell et al. 2002).

Five studies measured vitamin E in the third trimester. One study reported it was significantly lower (Saker et al. 2008), one reported it was nonsignificantly higher (Ortega-Senovilla et al. 2010), and one study reported it was significantly higher for SGA vs. appropriate for gestational age (AGA) births (Schiff et al. 1996). Interestingly, the two studies with serial measures showed that lipid-adjusted α-tocopherol was not significantly different during the second trimester but became significantly lower in SGA vs. AGA at the start of the third trimester (28 weeks) (Scholl et al. 2006; Chappell et al. 2002).

γ-Tocopherol and SGA (4 studies)

No additional details

Vitamin C

Vitamin C and preeclampsia

First/Second Trimester (3 studies)

One study measured levels of vitamin C in the first trimester and found lower levels in women who subsequently developed preeclampsia (Zhang, Luthy, et al. 2001); the adjusted OR was 3.1 (95% CI: 1.1-9.4) for those below the 10th percentile for vitamin C. Two studies measured vitamin C in the second trimester. One found similar levels for cases and controls at 14-22 weeks (Rajasingam et al. 2009), and the other, which assessed vitamin C every four weeks from 20 weeks, found significantly lower vitamin C at every time point except 24 weeks (Chappell et al. 2002).

Third Trimester (30 studies)

One additional study reported that vitamin C levels were significantly lower among preeclampsia cases (Hubel et al. 1997). Another study reported that the levels were nonsignficantly lower among mild preeclampsia cases (Zusterzeel et al. 2002).

Vitamin C and SGA (3 studies)

Three studies examined the association between vitamin C levels in pregnancy and birth of an SGA infant. Two studies took samples during the second trimester and reported

significantly lower levels among pregnant women with subsequent SGA birth (Rajasingam et al. 2009; Chappell et al. 2002). Two of these studies measured levels in the third trimester and also found lower vitamin C levels among cases (Chappell et al. 2002; Saker et al. 2008). In the one study with serial measurements, taken every 4 weeks from 20 weeks, women with SGA birth had significantly lower vitamin C levels than controls at every assessment (Chappell et al. 2002). It should be noted, however, that cases were women with SGA birth who were also at high risk for preeclampsia, and controls were women with AGA births and at low-risk for preeclampsia, so there may well have been confounding by baseline risk.

Vitamin A/Retinol

Retinol and preeclampsia

First/Second Trimester (2 studies)

One study measured retinol in each trimester of pregnancy among women with diabetes and similar found levels at each timepoint between women who developed preeclampsia and those who did not (Azar et al. 2011). Another study measured retinol levels in the second trimester and also found that levels were similar between PE cases and controls (Rajasingam et al. 2009).

Third Trimester (13 studies)

No additional details

Retinol and SGA (6 studies)

No additional details

Carotenoids

Total carotene and preeclampsia (5 studies)

Additionally, Dirican et al. (2008) found total carotene to be significantly lower in the third trimester for both mild and severe preeclampsia cases vs. controls. Serdar et al. (2003) reported a statistically significant OR of 7 for severe cases in the lowest versus highest tertile of total carotene in the third trimester; and all other ORs were above 1 but not significantly so.

β-carotene and preeclampsia (9 studies)

Among those we were unable to pool, Azar et al. (2001) found β-carotene was lower in preeclampsia cases in the first and third trimesters; the difference was not statistically significant in the second trimester. After adjusting for confounding (BMI, HDL cholesterol, prandial status), the difference was significant only in the third trimester, and results were consistent for lipid-corrected β-carotene (total lipids). A published abstract by Wei et al. (2011) reported an adjusted OR for the lowest vs. the highest quartile of 6.0 (3.1, 11.9) at 24-26 weeks (adjustment variables

unclear from abstract). However, in two large case-control studies, neither reported any crude or adjusted ORs across quartiles significantly different from 1, nor did they suggest any consistent trend across quartiles (Zhang, Williams, et al. 2001; Williams et al. 2003).

α-carotene and preeclampsia (4 studies)

Azar et al. (2001) could not be pooled and found significantly lower levels in the third trimester, even after values were corrected for total lipids; however, no association was observed in the first or second trimester. Crude and adjusted ORs were not significantly different from 1 in either study that reported ORs (Zhang, Williams, et al. 2001; Williams et al. 2003).

Lycopene and preeclampsia (5 studies)

Azar et al. (2001) found that lycopene was higher in the second, but not in the first or third trimester, for cases of preeclampsia vs. controls, even after adjusting for confounding. Zhang, Williams et al. (2001) reported ORs by quartile; crude ORs were not significantly different from 1 but all were below 1 (reference is lowest level) suggesting that higher levels could be protective; however, the adjusted ORs suggested the opposite. Adjustment variables included total cholesterol.

Other carotenoids and preeclampsia (4 studies)

No additional details

Carotenoids and SGA (1 study)

No additional details

Appendix G: Odds ratios reported in the reviewed studies for vitamin E and preeclampsia

Xu 2011<br>12-18 weeks	Zhang 2001<br>36.8 ± 3.8 (cases and controls combined)	Williams 2003<br>37.4 ± 3.4 (cases and controls combined)	Serdar 2003<br>31-38 weeks
By quartile, lowest=reference<br><br>Total tocopherol<br>Q2 OR = 1.30 (0.67, 2.52)<br>Q3 OR = 1.13 (0.54, 2.37)<br>Q4 OR = 1.45 (0.65, 3.23)<br>z-score OR = 1.11 (0.85, 1.46)<br><br>Q2 AOR = 1.60 (0.78, 3.27)[1]<br>Q3 AOR = 1.17 (0.54, 2.53)<br>Q4 AOR = 1.34 (0.58, 3.08)<br>z-score AOR = 1.11 (0.83, 1.49)<br><br>a-tocopherol<br>Q2 OR = 0.96 (0.50, 1.83)<br>Q3 OR = 0.90 (0.44, 1.85)<br>Q4 OR = 1.06 (0.49, 2.32)<br>z-score OR = 1.05 (0.81, 1.37)<br><br>Q2 AOR = 1.07 (0.54, 2.11)[1]<br>Q3 AOR = 0.10 (0.48, 2.14)<br>Q4 AOR = 1.00 (0.44, 2.24)<br>z-score AOR = 1.06 (0.79, 1.42)<br><br>g-tocopherol<br>Q2 OR = 1.34 (0.65, 2.76)<br>Q3 OR = 1.02 (0.65, 2.76)<br>Q4 OR = 2.00 (0.95, 4.23)<br>z-score OR = 1.48 (1.13, 1.92)<br><br>Q2 AOR = 1.24 (0.58, 2.64)[1]<br>Q3 AOR = 1.00 (0.46, 2.10)<br>Q4 AOR = 1.63 (0.75, 3.37)<br>z-score AOR = 1.35 (1.02, 1.78)<br><br>g-/a-tocopherol ratio<br>Q2 OR = 1.10 (0.58, 2.09)<br>Q3 OR = 0.80 (0.41, 1.59)<br>Q4 OR = 1.88 (0.94, 3.76)<br>z-score OR = 1.52 (1.16, 2.00)<br><br>Q2 AOR = 1.08 (0.56, 2.10)<br>Q3 AOR = 0.80 (0.39, 1.67)<br>Q4 AOR = 1.39 (0.71, 3.10)<br>z-score AOR = 1.43 (1.08, 1.90)	By quartile, lowest=reference<br><br>g-tocopherol<br>Q2 OR = 0.75 (0.38, 1.49)<br>Q3 OR = 1.13 (0.59, 2.16)<br>Q4 OR = 1.30 (0.69, 2.45)<br><br>Q2 AOR = 0.47 (0.20, 1.08)[2]<br>Q3 AOR = 1.30 (0.61, 2.77)<br>Q4 AOR = 1.27 (0.59, 2.71)<br><br>a-tocopherol<br>Q2 OR = 1.24 (0.61, 2.52)<br>Q3 OR = 1.26 (0.62, 2.54)<br>Q4 OR = 2.38 (1.23, 4.60)<br><br>Q2 AOR = 1.71 (0.75, 3.93)[2]<br>Q3 AOR = 1.83 (0.70, 4.75)<br>Q4 AOR = 4.98 (1.77, 13.98)<br><br>Q2 AOR = 1.43 (0.61, 3.34)[3]<br>Q3 AOR = 1.22 (0.45, 3.32)<br>Q4 AOR = 3.13 (1.06, 9.23)<br><br>a-tocopherol/total cholesterol<br>Q2 OR = 1.69 (0.82, 3.38)<br>Q3 OR = 1.44 (0.68, 3.02)<br>Q4 OR = 2.88 (1.22, 5.57)<br><br>Q2 AOR = 1.73 (0.76, 3.92)[4]<br>Q3 AOR = 1.85 (0.81, 4.24)<br>Q4 AOR = 3.47 (1.60, 7.57)<br><br>a-tocopherol/total lipids<br>Q2 OR = 1.29 (0.66, 2.54)<br>Q3 OR = 1.28 (0.65, 2.52)<br>Q4 OR = 1.63 (0.84, 3.13)<br><br>Q2 AOR = 1.49 (0.69, 3.19)[6]<br>Q3 AOR = 1.57 (0.73, 3.35)<br>Q4 AOR = 2.16 (1.03, 4.52)	By quartile, lowest=reference<br><br>g-tocopherol<br>Q2 OR = 1.68 (0.92, 3.12)<br>Q3 OR = 1.32 (0.70, 2.50)<br>Q4 OR = 2.26 (1.24, 4.14)<br><br>Q2 AOR = 1.43 (0.68, 2.98)[5]<br>Q3 AOR = 0.97 (0.45, 2.08)<br>Q4 AOR = 1.44 (0.68, 3.06)<br><br>a-tocopherol<br>Q2 OR = 1.16 (0.58, 2.31)<br>Q3 OR = 1.78 (0.93, 3.42)<br>Q4 OR = 3.69 (1.99, 6.82)<br><br>Q2 AOR = 1.10 (0.50, 2.46)[6]<br>Q3 AOR = 1.15 (0.52, 2.56)<br>Q4 AOR = 1.65 (0.75, 3.60)	By tertile, highest=reference<br><br>T1 OR mild = 3.6 (0.9, 14.9)<br>T2 OR mild = 1.9 (0.6, 6.1)<br><br>T1 OR severe = 6.6 (1.6, 27.7)<br>T2 OR severe = 2.0 (0.6, 7.4)

1. Adjustment variables: smoking, the presence of pre-selected clinical risk condition (i.e. chronic hypertension, history of preeclampsia, diabetes), prenatal regular using of vitamins or mineral supplementation, intervention status (vitamins supplementation vs placebo), gestational age and baseline BMI

2. Adjustment variables: maternal age, nulliparity, prepregnancy body mass index (quartile), use of prenatal vitamins, gestational age at blood collection, education, planned pregnancy, and total cholesterol concentration
3. Adjustment variables: maternal age, nulliparity, prepregnancy body mass index (quartile), use of prenatal vitamins, gestational age at blood collection, education, planned pregnancy, and total lipid concentration (2 x cholesterol + triglycerides)
4. Adjusted for maternal age, nulliparity, prepregnancy body mass index (quartile), use of prenatal vitamins, gestational age at blood collection, education, and planned pregnancy
5. Adjustment variables: maternal age (<19; 19–34; and ≥35 years), nulliparity (yes/no), maternal adiposity, midarm circumference (continuous), gestational age (continuous), and prenatal vitamin use (yes/no)
6. Adjustment variables: maternal age (<19; 19–34; and ≥35 years), nulliparity (yes/no), maternal adiposity, midarm circumference (continuous), gestational age (continuous), prenatal vitamin use (yes/no), and plasma total triglycerides (quartile)

Appendix H: Results of influence analyses (for meta-analyses with 10 or more observations)

Meta-Analysis	Results of Influence Analysis
Vitamin A, All PE	None of these studies were highly influential on the overall result; however, the Jendryczko 1989 study was an outlier and the confidence interval did not overlap with any of the others; SMD -4.35 (-6.02, -2.68)
Vitamin C, All PE	Howlader 2007 and Nilar 2009 were somewhat influential on the overall result; however, exclusion of neither study changed the overall conclusion
Vitamin C, Mild PE	Sharma 2006 study was influential on the overall result. It is the only study that found significantly higher levels of vitamin C in cases but mistakenly reported in the text that they found the levels were lower. When we omitted this study from the meta-analysis, we obtained a significantly negative pooled SMD; -0.43 (-0.69, -0.18).
Vitamin C, Severe PE	None of these studies were very highly influential on the overall result. Exclusion of Sharma 1984, Islam 2004, Noyan 2006, or Sharma 2006 studies would have resulted in a significantly negative pooled SMD.
Vitamin E, All PE	None of these studies were highly influential on the overall result
Vitamin E, Mild PE	Kaur 2008 study was influential on the overall result. Omitting this study from the meta-analysis resulted in a narrower confidence interval and a pooled SMD -0.18 (-0.54, 0.18). However, exclusion of this study would not change the overall conclusion of no significant difference. The Akyol 2000 study was also somewhat influential in the opposite direction, but did not impact the conclusion of no difference.
Vitamin E, Severe PE	None of these studies were highly influential on the overall result
Lipid-Adjusted Vitamin E, All PE	None of these studies were highly influential on the overall result

Appendix I: Tables from additional meta-regression analyses

α-tocopherol & All Preeclampsia (N overall=15; SMD= -0.35, 95% CI -0.66,-0.03, I^2=88%)

Covariate	No.	β-coef	95% CI	P	I^2, %	Adj. R^2, %
Univariate models						
Prospective design	1					
Study Quality						
Addressed Confounding	10	0.85	0.23, 1.46	0.01	78.7	48.34
NOS>4.5 (above median)	7	0.47	-0.24, 1.18	0.18	84.3	10.05
Matched	4	0.28	-0.57, 1.14	0.48	88.5	-3.41
Population / Setting						
General Population	13	0.23	-0.95, 1.40	0.68	89.0	-7.30
High-risk for preeclampsia	2	-0.23	-1.40, 0.95	0.68	89.0	-7.30
LMIC	8	-0.22	-0.98, 0.54	0.55	88.7	-9.15
Exposure Characteristics						
Fasting	8	-0.72	-1.34, -0.10	0.03	80.3	36.91
HPLC	13	1.06	0.10, 2.01	0.03	86.5	25.56
Pre-labor sample	6	-0.22	-1.00, 0.55	0.55	86.9	-4.45
MD gestational age (per wk)	13	-0.22	-0.49, 0.06	0.12	84.1	21.94
MD maternal age (per year)	15	-0.14	-0.42, 0.13	0.27	89.2	-2.47
MD BMI (per unit kg/m2)	7	-0.09	-0.51, 0.33	0.62	81.7	-23.11
Multivariable model intercept*	14	-0.85	-1.51, -0.20	0.02	63.4	76.81
HPLC		1.07	0.39, 1.76	0.01		
Fasting		-0.84	-1.30, -0.39	<0.01		

Vitamin E & Mild Preeclampsia (N overall=12; SMD= 0.09, 95% CI -0.55, 0.72, I^2=93%)

Covariate	No.	β-coef	95% CI	P	I^2, %	Adj. R^2, %
Univariate models						
Prospective design	1					
Study Quality						
Addressed Confounding	6	0.14	-1.59, 1.86	0.86	93.4	-10.57
NOS>4.5 (above median)	7	-1.00	-2.61, 0.59	0.19	93.0	8.40
Matched	3	0.14	-1.86, 2.15	0.88	93.3	-10.42
Population / Setting						
General Population	9	0.39	-1.58, 2.36	0.67	93.4	-8.52
High-risk for PE	2	-0.64	-2.89, 1.62	0.54	93.2	-6.22
LMIC	7	-0.06	-1.81, 1.69	0.94	93.8	-11.13
Exposure Characteristics						
Fasting	4	-0.46	-2.26, 1.34	0.58	93.2	-6.83
HPLC	9	1.18	-0.65, 3.01	0.18	93.3	8.33
Pre-labor sample	4	-0.26	-2.08, 1.56	0.75	93.5	-9.68
α-tocopherol	4	-0.35	-2.17, 1.46	0.67	93.4	-8.55
Definition includes adverse events	8	-0.13	-1.96, 1.70	0.88	93.8	-10.98
MD gestational age (per wk)	8	0.05	-0.51, 0.60	0.84	81.8	-22.92
MD maternal age (per year)	7	0.15	-0.40, 0.71	0.50	82.2	-12.60
MD BMI (per unit kg/m2)	0					
Multivariable model intercept*	11	-0.36	-1.89, 1.17	0.60	92.8	35.4
NOS>4.5 (above median)		-1.40	-2.96, 0.17	0.07		
HPLC		1.71	-0.05, 3.48	0.06		

Vitamin E & Severe Preeclampsia (N overall=17; SMD= -0.13 95% CI -0.57, 0.30, I^2=90%)

Covariate	No.	β-coef	95% CI	P	I^2, %	Adj. R^2, %
Univariate models						
Prospective design	1					
Study Quality						
Addressed Confounding	8	-0.13	-1.07, 0.82	0.78	90.2	-7.15
NOS>4.5 (above median)	8	-0.28	-1.22, 0.65	0.52	90.1	-4.43
Matched	5	-0.07	-1.11, 0.97	0.89	90.0	-7.61
Population / Setting						
General Population	12	0.12	-0.93, 1.16	0.81	90.2	-7.04
High-risk for PE	4	-0.11	-1.25, 1.02	0.84	90.2	-7.08
LMIC	12	-0.40	-1.41, 0.61	0.41	90.0	-2.39
Exposure Characteristics						
Fasting	5	-0.70	-1.67, 0.28	0.15	88.4	8.83
HPLC	12	0.54	-0.45, 1.54	0.26	89.0	3.35
Pre-labor sample	5	-0.19	-1.23, 0.84	0.70	90.1	-6.59
A-tocopherol	6	-0.74	-1.64, 0.17	0.10	87.3	13.13
Definition includes adverse events	10	-0.26	-1.21, 0.69	0.57	90.2	-5.27
MD gestational age (per wk)	13	0.05	-0.16, 0.25	0.62	83.1	-5.33
MD maternal age (per year)	12	0.13	-0.04, 0.31	0.12	78.2	17.16
MD BMI (per unit kg/m2)	4	0.27	-0.76, 1.30	0.38	78.5	15.44
Multivariable model intercept	12	-0.18	-0.60, 0.24	0.35	63.4	56.50
WMD maternal age (per year)		0.17	0.02, 0.31	0.03		
Fasting		-1.01	-1.90, -0.11	0.03		

Vitamin C & All Preeclampsia (N overall=29; SMD= - 0.56, 95% CI -0.83,-0.28, I^2=91%)

Covariate	No.	β-coef	95% CI	P	I^2, %	Adj. R^2, %
Univariate models						
Prospective design	1					
Study Quality						
Addressed Confounding	18	0.19	-0.62, 1.00	0.49	91.2	-3.39
NOS>4.5 (above median)	10	-0.66	-1.44, 0.12	0.09	90.5	7.94
Matched	9	0.21	-0.63, 1.05	0.62	90.8	-3.08
Population / Setting						
General Population	23	-0.25	-1.22, 0.72	0.60	91.2	-3.42
High-risk for PE	4	0.19	-0.95, 1.33	0.74	91.2	-4.00
LMIC	21	-0.26	-1.13, 0.62	0.55	91.2	-3.15
Exposure Characteristics						
Fasting	9	0.15	-0.70, 1.00	0.72	91.1	-4.11
HPLC	6	-0.01	-0.99, 0.96	0.98	91.1	-4.38
Pre-labor sample	9	-0.54	-1.37, 0.28	0.19	90.4	3.73
MD gestational age (per wk)	23	0.00	-0.25, 0.25	0.99	92.3	-5.47
MD maternal age (per year)	24	0.01	-0.26, 0.27	0.96	91.1	-5.36
MD BMI (per unit kg/m2)	7	0.09	-0.16, 0.34	0.40	64.5	3.65

For all tables in Appendix 9: *Restricted to prospective studies. CI, confidence interval; HPLC, high-performance liquid chromatography; LMIC, low- or middle-income country; MD, mean difference; NOS, Newcastle-Ottawa Scale [score]

Vitamin C & Mild Preeclampsia (N overall=11; SMD= -0.32, 95% CI -0.67, 0.03, I^2=77%)

Covariate	No.	β-coef	95% CI	P	I^2, %	Adj. R^2, %
Univariate models						
Prospective design	0					
Study Quality						
Addressed Confounding	6	-0.24	-1.02, 0.54	0.5	79.0	-8.05
NOS>4.5 (above median)	4	-0.69	-1.39, 0.00	0.05	68.9	35.51
Matched	3	0.27	-0.59, 1.13	0.49	78.7	-7.26
Population / Setting						
General Population	9	0.36	-0.64, 1.36	0.44	77.6	-3.03
High-risk for PE	1					
LMIC	7	0.42	-0.37, 1.20	0.26	76.0	6.07
Exposure Characteristics						
Fasting	5	-0.51	-1.19, 0.18	0.13	67.5	26.59
HPLC	3	-0.67	-1.46, 0.13	0.09	72.1	25.42
Pre-labor sample	3	-0.58	-1.37, 0.21	0.13	72.8	20.58
Definition includes adverse events	8	0.18	-0.70, 1.07	0.65	78.51	-9.15
MD gestational age (per wk)	6	0.13	-0.05, 0.31	0.11	0	
MD maternal age (per year)	6	0.06	-0.10, 0.23	0.36	0	
MD BMI (per unit kg/m2)	0					
Multivariable model intercept	11	0.12	-0.30, 0.54	0.55	55.1	58.10
NOS>4.5 (above median)		-0.63	-1.26, 0.00	0.05		
Fasting		-0.46	-1.05, 0.13	0.11		

Vitamin C & Severe Preeclampsia (N overall=15; SMD= -0.35, 95% CI -0.72, 0.01, I^2=84%)

Covariate	No.	β-coef	95% CI	P	I^2, %	Adj. R^2, %
Univariate models						
Prospective design	0					
Study Quality						
Addressed Confounding	7	0.08	-0.78, 0.95	0.84	83.6	-8.63
NOS>4.5 (above median)	4	-0.69	-1.57, 0.21	0.12	81.2	13.3
Matched	4	0.60	-0.29, 1.49	0.17	77.7	13.55
Population / Setting						
General Population	12	0.11	-1.00, 1.22	0.83	84.5	-8.66
High-risk for PE	2	0.02	-1.32, 1.36	0.98	84.8	-8.73
LMIC	11	0.29	-0.68, 1.25	0.53	84.9	-7.15
Exposure Characteristics						
Fasting	6	-0.24	-1.12, 0.65	0.57	83.1	-5.07
HPLC	4	-0.55	-1.50, 0.39	0.23	82.6	5.56
Pre-labor sample	3	-0.42	-1.48, 0.63	0.40	83.6	-2.52
Definition includes adverse events	9	-0.73	-1.50, 0.05	0.06	79.8	23.15
MD gestational age (per wk)	10	0.09	-0.14, 0.32	0.41	83.9	-3.64
MD maternal age (per year)	10	0.02	-0.32, 0.36	0.89	84.2	-16.26
MD BMI (per unit kg/m2)	3	0.11	-1.71, 1.92	0.59	56.6	-48.8
Multivariable model intercept	15	0.19	-0.41, 0.79	0.51	76.8	32.17
NOS>4.5 (above median)		-0.57	-1.40, 0.27	0.16		
Definition includes adverse events		-0.64	-1.40, 0.11	0.09		
