# Supplementary material for: Maternal Antioxidant Levels in Pregnancy and Risk of Preeclampsia and Small for Gestational Age Birth: A Systematic Review and Meta-Analysis
Source: PLoS One. 2015 Aug 6;10(8):e0135192. doi: 10.1371/journal.pone.0135192 (PMC4527773; doi:10.1371/journal.pone.0135192)
Supplement: S1 Protocol — (PDF) [file pone.0135192.s003.pdf]

UNIVERSITY of York
Centre for Reviews and Dissemination

NHS
National Institute for
Health Research

PROSPERO International prospective register of systematic reviews

Risk of preeclampsia and small for gestational age according to antioxidants levels throughout pregnancy

Jacqueline Cohen, Susan Kahn, Olga Basso, Robert Platt, Haim Abenhaim, Michael Kramer

Citation

Jacqueline Cohen, Susan Kahn, Olga Basso, Robert Platt, Haim Abenhaim, Michael Kramer. Risk of preeclampsia and small for gestational age according to antioxidants levels throughout pregnancy. PROSPERO 2013:CRD42013003519 Available from
http://www.crd.york.ac.uk/PROSPERO_REBRANDING/display_record.asp?ID=CRD42013003519

Review question(s)

To conduct a comprehensive systematic literature review to address the question of whether, among pregnant women, the levels of maternal antioxidants at different time points throughout pregnancy are associated with risk of preeclampsia or SGA.

Searches

Our search strategy will initially be developed in Medline and then adapted to the other databases, as described below. A number of other databases will be searched in order to identify relevant studies. We will assess our search strategy by its ability to identify records of known studies and we will further refine it after preliminary searches to include all relevant controlled vocabulary (indexing subject heading including MeSH terms in Medline and Emtree terms in Embase) and free text words in the title or abstract.

The final search strategy will include searches of MEDLINE, EMBASE, ProQuest Dissertations & Theses, CAB Abstracts, CINAHL, FSTA Direct, BIOSIS Previews, and POPLINE databases.

Search terms will include but are not limited to the following: The MeSH terms “antioxidants” [MeSH] (includes “tocopherols” [MeSH], “vitamin E” [MeSH]), “carotenoids” [MeSH], “vitamin A” [MeSH],“ascorbic acid”[MeSH], “preeclampsia” [MeSH], “infant, small for gestational age” [MeSH], and “fetal growth retardation”[MeSH] will be used. Other search terms that will be combined using appropriate Boolean operators include “antioxidant*”, “tocopherol*”, “vitamin E”, “carotenoid*”, “lycopene”, “cryptoxanthin,” lutein,” “vitamin A,” “retinol,” “vitamin C,” “ascorbic acid,” “ascorbate,” “preeclampsia,” “pre-eclampsia,” “toxemia,” “small for gestational age,” “SGA,” “fetal growth retardation,” “fetal growth restriction,” “FGR,” “intrauterine growth retardation,” “intra-uterine growth retardation,” “intrauterine growth restriction,” “intra-uterine growth restriction,” and “IUGR.”

Types of study to be included

We will include prospective longitudinal studies (including RCTs where antioxidants levels were measured in the placebo arm), nested case-control studies, case-control studies, and cross-sectional studies. We will include cross-sectional studies of mothers of SGA infants (as SGA can only be reliably diagnosed postpartum) that recruited women at the time of delivery.

Condition or domain being studied

Preeclampsia is a hypertensive disorder that affects 2-8% of pregnancies worldwide, and is most common in first pregnancies. Although it can be diagnosed from the 20th week of pregnancy, most cases occur near term. Preeclampsia is a poorly understood and heterogeneous disorder that is typically diagnosed by the presence of high blood pressure and protein in the urine. It is a multisystem disorder, which may affect the brain, lungs, kidney, and liver. Pregnant women with preeclampsia are at risk for severe complications including seizures (eclampsia), multi-organ failure, stroke, and death. Risks to the fetus include preterm birth, suboptimal growth resulting in small for gestational age (SGA) neonate, and stillbirth.

Small for gestational age (SGA) is defined as the birth of an infant at less than the 10th percentile of gestational-age-

and sex- specific-birth weight, according to a given population reference. Hence, SGA is often the result of intrauterine growth restriction (IUGR), also known as fetal growth restriction (FGR), but may also include some constitutionally small babies. The prevalence of SGA is, by definition, approximately 10% among live births. SGA shares a similar risk factor profile to preeclampsia and potentially shared underlying pathology.

Participants/ population

We are interested in pregnant women with preeclampsia or SGA risk factors such as pre-existing disease that increases preeclampsia or SGA risk, personal or family history of preeclampsia, or abnormal ultrasound findings, as well as in women taking part in population-based studies where the outcomes of SGA and preeclampsia were recorded.

Intervention(s), exposure(s)

We are interested in maternal blood levels of non-enzymatic antioxidant levels including tocopherols (vitamin E), carotenes (alpha-carotene, beta-carotene and lycopene), oxy-carotenoids (lutein, zeaxanthin, cryptoxanthin), retinol (vitamin A), and ascorbate (vitamin C). We will include studies of maternal antioxidant levels measured from samples obtained at any point before delivery, or samples taken shortly after delivery. We will not assess cord blood levels or maternal samples taken >72 hours after delivery. However, we believe that our search strategy will capture these studies, which will enable us to briefly summarize them in a section on excluded studies. We will exclude studies published prior to 1970, when high-performance liquid chromatography technology used for precise measurement of many antioxidant biomarker levels was developed.

Comparator(s)/ control

Control subjects will be pregnant women who did not develop preeclampsia of fetal growth restriction in the course of pregnancy or deliver a small for gestational age baby.

Outcome(s)

Primary outcomes

Primary outcomes of interest include preeclampsia and SGA or intrauterine/fetal growth restriction documented by serial ultrasound scans.

Secondary outcomes

Secondary outcomes include severe preeclampsia (including eclampsia), early-onset preeclampsia (onset

PROSPERO
International prospective register of systematic reviews
The information in this record has been provided by the named contact for this review. CRD has accepted this information in good faith and registered the review in PROSPERO. CRD bears no responsibility or liability for the content of this registration record, any associated files or external websites.
